# Parkinson-causing α-synuclein missense mutations shift native tetramers to monomers as a mechanism for disease initiation

**DOI:** 10.1038/ncomms8314

**Published:** 2015-06-16

**Authors:** Ulf Dettmer, Andrew J. Newman, Frank Soldner, Eric S. Luth, Nora C. Kim, Victoria E. von Saucken, John B. Sanderson, Rudolf Jaenisch, Tim Bartels, Dennis Selkoe

**Affiliations:** 1 Ann Romney Center for Neurologic Diseases, Department of Neurology, Brigham and Women's Hospital and Harvard Medical School, Boston, Massachusetts 02115, USA; 2 The Whitehead Institute, Cambridge, Massachusetts 02142, USA; 3 Department of Biology, Massachusetts Institute of Technology, Cambridge, Massachusetts 02139, USA

## Abstract

β-Sheet-rich α-synuclein (αS) aggregates characterize Parkinson's disease (PD). αS was long believed to be a natively unfolded monomer, but recent work suggests it also occurs in α-helix-rich tetramers. Crosslinking traps principally tetrameric αS in intact normal neurons, but not after cell lysis, suggesting a dynamic equilibrium. Here we show that freshly biopsied normal human brain contains abundant αS tetramers. The PD-causing mutation A53T decreases tetramers in mouse brain. Neurons derived from an A53T patient have decreased tetramers. Neurons expressing E46K do also, and adding 1-2 E46K-like mutations into the canonical αS repeat motifs (KTKEGV) further reduces tetramers, decreases αS solubility and induces neurotoxicity and round inclusions. The other three fPD missense mutations likewise decrease tetramer:monomer ratios. The destabilization of physiological tetramers by PD-causing missense mutations and the neurotoxicity and inclusions induced by markedly decreasing tetramers suggest that decreased α-helical tetramers and increased unfolded monomers initiate pathogenesis. Tetramer-stabilizing compounds should prevent this.

## Abstract (web summary)

ß-Sheet-rich aggregates of α-synuclein (αS) characterize Parkinson's disease (PD) and recent work suggests that αS exists as α-helix-rich tetramers and related low-*n* multimers in normal cells. Here the authors show that αS mutations associated with familial PD decrease the intraneuronal tetramer:monomer ratio and are correlated with neurotoxicity.

## Graphical abstract

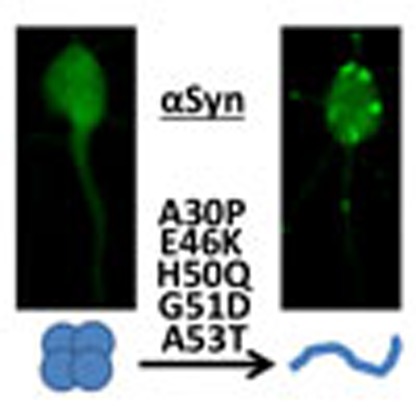

α-Synuclein (αS) accumulates as insoluble protein aggregates in Lewy bodies and Lewy neurites, the hallmarks of familial and sporadic Parkinson's disease (PD) and several other fatal diseases^1^. αS missense mutations, copy number variants and upregulated expression have each been associated with familial PD^2^,^3^,^4^,^5^,^6^. Unexpected findings from our^7^ and one other laboratory^8^ suggested that αS can occur physiologically not only as unfolded monomers but in large part as multimers, principally tetramers, that have α-helical conformation and resist aggregation. Crosslinking analyses of endogenous αS in living cells^9^ supported this new model: in primary neurons and other cells, cell-penetrant crosslinkers disuccinimidyl glutarate (DSG) or dithiobis(succinimidyl propionate) (DSP) trapped αS in abundant species larger than monomer, especially at ∼60 kDa, the size of four *N*-acetylated αS monomers (4 × 14,502 daltons=58,010 daltons). Known monomeric proteins (for example, cytosolic tubulin; Parkin; Ran) were not altered by the crosslinking, while known multimeric proteins (for example, DJ-1; voltage-dependent anion channel (VDAC); dynamin-related protein 1 (Drp1)) were trapped as the expected multimers. We systematically ruled out artifactual induction of these multimers by the crosslinking, abnormally migrating monomers, and hetero-oligomers with other proteins^9^. In addition to the major ∼60 kDa apparent tetramer (αS60), smaller amounts of endogenous αS were trapped as multimers of ∼80 and ∼100 kDa (αS80 and αS100) that have the same isoelectric point as the monomer and may be conformers of the tetramer or higher-order multimers (for example, hexamer and octamer)^9^. All multimers were depolymerized to monomers when crosslinkers were applied after cell lysis, unless the lysis was done at high protein concentrations (‘molecular crowding')^9^. Other endogenous multimeric proteins did not show this striking cell lysis sensitivity, except for the homolog β-synuclein (βS)^9^. These findings led us to postulate that dynamic intracellular populations of αS monomers and metastable multimers co-exist normally^7^,^9^. Such a dynamic equilibrium has been proposed for well-known tetrameric proteins such as p53 (refs 10, 11) and haemoglobin^12^.

Some labs subsequently published data they interpreted as supporting the earlier model of αS existing as natively unfolded monomers in cells and brain. However, these studies either did not employ crosslinking of intact cells^13^ or considered any crosslinked oligomeric αS to be non-specific^14^. The possiblilty that the endogenous multimers we trap by crosslinking are pathological is made highly unlikely by biophysical evidence of α-helical tetramers (∼58 kDa) purified natively from normal human cells^7^,^15^, the abundance of trapped tetramers in healthy, non-transfected cells^9^,^16^ and the trapping in analogous tetrameric forms of the non-pathogenic βS and of αS lacking its aggregation-prone NAC domain^9^.

This unresolved but central debate about the existence and biological relevance of αS tetramers led us to use additional approaches. Here we first examined αS in freshly biopsied normal human brain, the most physiologically relevant source of the protein. To do so, we developed a method for intact-cell crosslinking of fresh brain tissue that reveals the endogenous state of αS. Second, we asked whether αS missense mutations that unequivocally cause PD might alter the tetramer:monomer ratio in human neurons. Despite almost two decades of study, the fundamental mechanism by which these mutants initiate αS aggregation, Lewy body formation and PD remains unclear. To this end, we rigorously standardized our intact-cell crosslinking method until it was precise enough to quantify accurately the effects of each of the five PD-causing αS missense mutations. And third, we applied fluorescent protein complementation to independently confirm our key findings in living cells. Using both live-cell methods (chemical crosslinking and yellow fluorescent protein (YFP) complementation), we found that PD-causing αS missense mutations partially shifted cellular αS from tetramers/multimers to monomers. This shift became prounced when we amplified the effect of the E46K PD mutation by making similar E-to-K substitutions in two adjacent KTKEGV repeat motifs, leading to marked reduction of cellular tetramers, decreased αS solubility, formation of round inclusions and cytoxicity.

## Results

### Abundant αS tetramers occur in normal human and mouse brain

**Figure 1:**
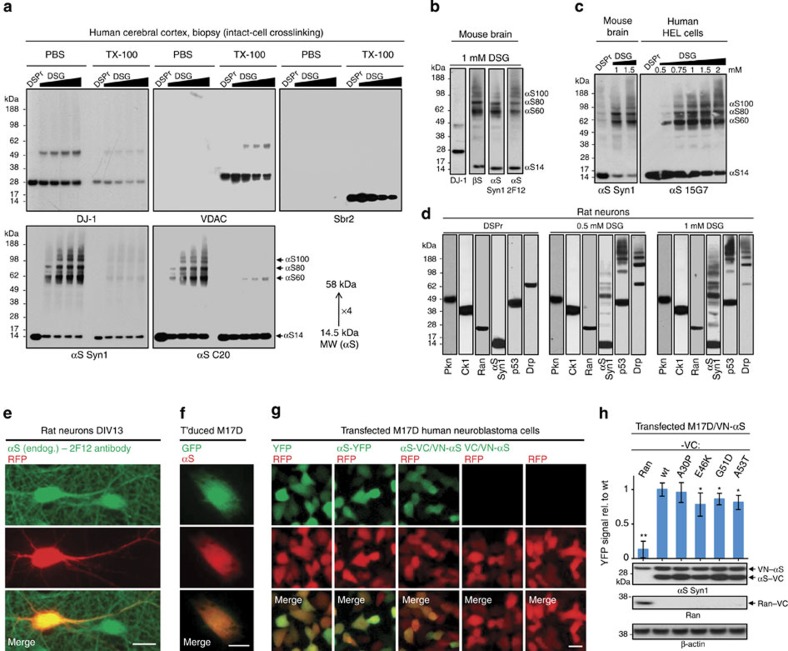
αS multimers in normal brain tissues and neural cells. (**a**) A fresh human cortical biopsy was crosslinked with either 1.75 mM DSP and reduced by βME (DSPr) or else with 1 mM DSG at increasing volume-to-protein ratio, followed by sequential extraction of PBS- and TX-100-soluble fractions. Each lane is one technical replicate from the biopsy. (**b**) Mouse brain, 1 mM DSG, PBS fraction; blots represent five independent experiments from different WT mice. (**c**) Mouse brain and human erythroleukemia (HEL) cells. DSG concentration gradients applied as indicated, and PBS fractions prepared; blots represent at least three independent experiments. (**d**) DIV13 rat neurons. 1.75 mM DSP/βME (DSPr, left panel), 0.5 mM DSG (middle) and 1 mM DSG (right) were applied, and PBS fractions prepared. Western blots for αS (Syn1 mAb), the monomeric proteins Parkin (Pkn), casein kinase 1α (CK1) and Ran, and the tetrameric proteins p53 and Drp1 (Drp); blots represent at least 3 independent experiments from different primary cultures. (**e**) Fluorescence microscopy of DIV13 rat neurons: endogenous αS in green (mAb 2F12) and transfected RFP (red), plus merged image; scale bar, 20 μm. (**f**) Fluorescence microscopy of virally transduced M17D cells: αS in red (mAb 2F12), GFP in green; scale bar, 5 μm. (**g**) Fluorescence microscopy of M17D cells transiently transfected with RFP plus YFP or αS-YFP or YFP αS complementation pairs, as indicated (empty vector control on far right). YFP in green, RFP in red, merge below; scale bar, 10 μm. (**h**) Quantification of multiple Venus-YFP complementation assays. A stable cell line M17D/VN-αS was transfected with DNA constructs expressing VC fused to αS (WT, A30P, E46K, G51D or A53T) or Ran (negative control). YFP complementation intensity relative to WT αS-VC transfection from N=8 independent experiments on 4 days using different DNA preparations;*P<0.05, **P<0.01, Student's *t*-test; relative to WT in this and subsequent figures, unless stated otherwise; error bars, s.d. Below are representative western blots for αS (Syn1 mAb), Ran, and the loading control β-actin.

We previously reported that the crosslinking pattern of endogenous αS in intact cells differed from the stochastic crosslinking of recombinant monomeric αS *in vitro*, in that the former strongly favoured the trapping of tetramers, with few or no intermediate dimers or trimers^9^. We now sought to crosslink αS in its endogenous state in normal brain tissue. We achieved this by mincing fresh brain with a mechanical tissue chopper, performing gentle centrifugal washes on the intact tissue pieces and then subjecting only these washed brain bits to crosslinking, thereby largely avoiding the tetramer-destabilizing effects of breaking cells open^9^ (see Methods and Supplementary Fig. 1). We applied this protocol to an optimal source of physiological αS: a fresh brain biopsy (elective surgery for focal epilepsy) from the cerebral cortex of a young patient free of any neurodegenerative process. After the mincing and centrifugal washes, we applied DSG (1 mM solution) at an increasing ratio of volume of crosslinking solution to brain protein, or else applied the reducible crosslinker DSP (2 mM solution) at a fixed ratio. The crosslinked tissue bits were then sonicated and separated (100,000 *g* spin) into PBS (‘cytosol') and Triton X-100 (TX-100; ‘membrane') fractions. Immunoblotting confirmed efficient crosslinking had occurred, as the cytosolic protein DJ-1 and the transmembrane protein VDAC were each trapped in their native dimeric states in a DSG dose-dependent manner (Fig. 1a, upper panels). As before, we fixed blots in paraformaldehyde^17^ to improve αS retention during blot washing^16^. Total αS levels were compared using the DSP-crosslinked-and-reduced sample, which we had shown previously was optimal for quantifying total cellular αS levels in crosslinking studies^16^. Enrichment of DJ-1 and absence of VDAC and synaptobrevin-2 established cytosol purity (Fig. 1a, upper panels). Immunoblotting confirmed our earlier findings in cultured neurons^9^: αS was detected in the biopsied human brain primarily as tetramers (αS60) and related multimers (αS80, αS100) by mAbs Syn1 and 15G7 (Fig. 1a). Antiserum C20 preferentially detected monomers, although its αS60:14 ratio was still 1:1 versus 2:1 using mAb Syn1 (Fig. 1a, lower panels). Including the αS80 and αS100 multimers with the αS60 tetramers showed the clear preponderance of αS multimers over monomers (3:1) in normal human brain. Similar to our findings in primary neurons^9^, the multimers were found overwhelmingly in the human brain cytosol (Fig. 1a, lower panels). In the membrane fraction, virtually only monomer was detected.

Next, we obtained closely similar results in minced fresh wild-type (WT) mouse brain using antibodies to αS and, importantly, to βS (Fig. 1b). Applying increasing DSG concentrations to mouse brain bits, human erythroleukemia (HEL) cells (Fig. 1c) or cultured primary neurons (Fig. 1d) did not completely trap αS at discrete multimeric positions. Instead, αS60 levels plateaued at a certain DSG concentration; further depletion of free 14 kDa monomer only added to high molecular weight (HMW) smears of αS immunoreactivity, probably the result of non-specific overcrosslinking (Fig. 1c). Similar to αS, known tetrameric proteins such as p53 (refs 18, 19) and Drp1 (ref. 20) exhibited HMW smears as well as oligomeric bands higher than their expected tetramer positions, especially at high DSG (Fig. 1d). Interestingly, crosslinking of the known tetrameric protein p53 was similar to αS in that intermediate dimers were of low abundance, whereas Drp1 showed a more ladder-like oligomer pattern (Fig. 1d). Monomeric cytosolic proteins such as Parkin, Ran and casein kinase 1α were always unaffected by crosslinking, as expected (Fig. 1d; Supplementary Fig. 2 for uncropped blots). Compared with the known tetrameric proteins, crosslinking of the known dimeric proteins DJ-1 or VDAC led to less HMW smears (Fig. 1a). These multiple control proteins support the ability of our crosslinking protocol to detect endogenous tetramers of proteins like p53, αS and βS in intact cells and brain tissue and not to induce artifactual oligomers of known monomeric proteins.

These data support a model in which αS exists predominantly as cytosolic multimers at steady state, while a smaller cytosolic portion and most αS on membranes (together representing a minority of total cellular αS) appears to be monomeric. To address the localization of intracellular αS without disrupting the cell membrane, we performed immunofluorescence microscopy of endogenous αS in primary rat neurons (Fig. 1e) and also virally expressed WT αS in human M17D neuroblastoma cells, which have very low endogenous αS (Fig. 1f). In both cell types, αS strongly co-localized with transiently expressed red fluorescent protein (RFP; Fig. 1e) or green fluorescent protein (GFP; Fig. 1f), which are known to be soluble and cytoplasmic. Next, we transfected M17D cells with αS fused to either intact YFP (Fig. 1g, second column) or else split YFP, that is, we co-transfected VN-αS (N-terminal half of Venus-YFP fused to N-terminus of an αS monomer) and αS-VC (C-terminal half of Venus-YFP fused to the C-terminus of an αS monomer) (Fig. 1g, third column). YFP complementation assays monitor the interaction of intracellular proteins in real-time and are based on the principle that interactions of the tagged protein (here αS) bring VN and VC into close proximity, leading to Venus-YFP complementation and thus fluorescence detectable by microscopy or plate reader. The living cells showed strong YFP fluoresence that co-localized with free cytosolic RFP and was distributed similarly to YFP alone (Fig. 1g, first column). These controls confirmed previously reported fluorescent complementation of WT αS using such an assay^21^ and underscored the cytosolic nature of these multimeric species. VC alone (C-terminal half of Venus-YFP) did not complement with VN-αS, ruling out an intrinsic affinity of VC and VN as responsible for the observed complementation (Fig. 1g: fourth versus fifth columns).

### Two independent methods to quantify cellular αS multimers

To standardize the fluorescent complementation assay for repeated measurements, we generated a VN-αS stable M17D line, enabling highly consistent complementation signals by transiently transfecting this line with various WT or mutant αS-VC constructs. Using an automated plate reader to quantify YFP fluorescence, we found that VN-αS complementation with VC-tagged Ran (a control cytosolic protein) was negligible compared with αS-VC (quantified in Fig. 1h: first two lanes). Additional controls for signal specificity, including the need for specific fusion positions of αS relative to the fluorescent protein fragments and the negligible self-complementation of the split YFP fragments alone (see Fig. 1g, fourth lane), were reported previously for αS GFP-complementation^22^ and YFP-complementation^21^. Despite the rapid and strong self-interaction we detected for WT αS (Fig. 1h, second lane) and the widespread use of fluorescent protein complementation to study native protein interactions, the αS field has heretofore interpreted such complementation as indicative of pathological oligomerization, given the long-standing assumption that the normal state of cellular αS is monomeric^14^. We analysed the effects of the known familial PD-linked missense mutations and observed significant reductions of fluorescent complementation for the E46K, G51D and A53T mutants versus WT αS (Fig. 1h). A30P complementation was similar to WT, but we consistently observed increased expression of A30P and also G51D by immunoblotting (Fig. 1h; top western blot), indicating that these two variants lead to enhanced αS-VC levels, and thus normalized values might detect reduced complementation for A30P as well. The decreased αS complementation seen here with three of the four familial PD (fPD) mutants studied supports the physiological nature of the multimers (otherwise, fPD mutations should increase complementation). There are certain disadvantages of this system: αS is modified by large tags; determining the sizes of the multimers is not possible; YFP complementation may be irreversible^23^ and thus disturb multimer:monomer equilibria; and crosslinking trapped abundant dimers but less tetramers of VN-αS+αS-VC (Supplementary Fig. 3), a pattern different from endogenous (untagged) αS. The latter result may be due to the relative steric hindrance we previously reported for tagged αS^9^ and/or decreased access by the crosslinker into any tagged tetramers.

**Figure 2:**
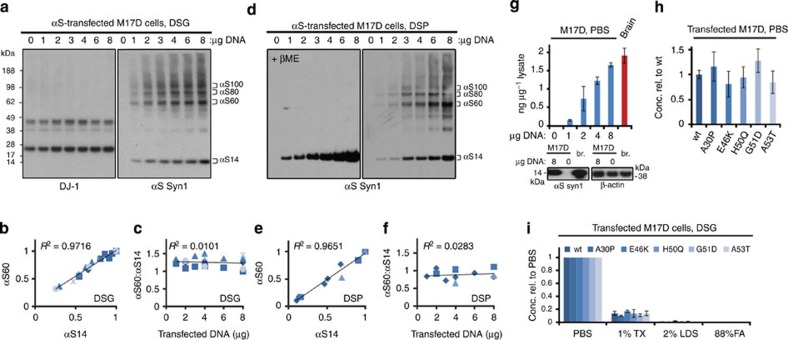
Intact-cell crosslinking of αS expressed at varying levels. (**a**) DSG-crosslinking analysis of an αS DNA gradient (0-8 μg per 6-cm culture dish). Western blots for αS (Syn1) and endogenous DJ-1. (**b**) DSG samples: αS60 intensities plotted against αS14 (densitometry). Highest value in each series was set to 1; graph shows mean data for N=5 independent experiments of 1, 2, 3, 4, 6, 8 μg DNA (2 exps.), 2, 4, 8 μg DNA (1 exp.) and 4, 8 μg DNA (2 exps.); data points generated in the same experiment are indicated by identical symbols. (**c**) αS60:αS14 ratios for the same samples as in 2b. (**d**) DSP-crosslinking analysis of an αS DNA gradient (0; 1-8 μg); western blots (Syn1) for αS in non-reduced and βME-reduced samples (**e**) Quantification of DSP samples: αS60 versus αS14. Highest value in each series was set to 1; N=3 independent experiments of 1, 2, 3, 4, 6, 8 μg DNA (1 exp.), 2, 4, 8 μg (1 exp.) and 4, 8 μg (1 exp.). (**f**) αS60:14 ratios for the same samples as in (**e**). (**g**) ELISA analysis of αS DNA gradients (1, 2, 4, 8 μg) transfected into M17D cells compared to human cortical homogenate (PBS fraction). Below: western blots for 0 and 8 μg DNA transfection versus human brain homogenate (red). *N*=2 for gradients (different days, different DNA preps) and brain homogenates. (**h**) ELISA of αS WT and fPD mutant transfectants (8 μg per 6-cm dish), PBS fraction. Graph: concentrations versus αS WT set to 1 (*N*=10 independent transfections on 4 different days using at least 4 different DNA preps per αS variant). tf'ed, transfected. (**i**) ELISA of αS WT and fPD mutant transfectants after 1 mM DSG crosslinking and sequential extraction (PBS→PBS/1%Triton→2% LDS→88% formic acid=FA). Graph: concentrations relative to the PBS fractions of the respective αS variant set to 1.

In light of these disadvantages of relying solely on YFP complementation for αS multimer quantification, we sought to rigorously standardize our intact-cell crosslinking protocol as a second independent method (Methods). We transfected M17D cells with increasing amounts of WT αS cDNA and applied a constant DSG amount at 40 hr post transfection. By confirming equal DJ-1 dimer:monomer ratios in the same cytosols as an internal standard for crosslinking efficiency in all experiments (Fig. 2a, left panel), we observed a constant αS60:14 ratio across the range of αS expression levels we tested (Fig. 2a, right panel). Plotting the relative intensities of αS60 and αS14 in multiple independent experiments revealed a highly linear relationship of the two (*R*^2^=0.97) (Fig. 2b), and the apparent αS60:14 ratio (using Syn1 for detection) was a constant 1.2 in this series of experiments (Fig. 2c). (For the three multimer species combined (αS60+80+100), their ratio to monomer was 2.8.) We next tested the reducible crosslinker DSP (Fig. 2d) and again found a highly linear correlation (*R*^2^=0.97) between αS60 and αS14 levels (Fig. 2e), here with a constant ratio of 0.9 (Fig. 2f). Analysis of αS80 and αS100 revealed a similarly linear ratio to monomer across this range of αS expression (Supplementary Fig. 4), but we focused our further analyses on the αS60 tetramer as our standard readout for four reasons: an independent biophysical method (analytical ultracentrifugation) originally revealed that the principal α-helical multimer purified from fresh human erythrocytes under non-denaturing conditions has a molecular weight of 58 kDa, exactly that of four monomers^7^; all of our many intact-cell crosslinking experiments to date identified αS60 as the most abundant and consistent cellular multimer^7^,^9^,^16^; mass spectrometry confirmed here that αS60 contains only αS (Supplementary Fig. 5); and for quantification, αS60 is representative of all physiological αS multimers we detect (for example, Supplementary Fig. 4). The constant αS60:14 ratio across a wide range of cellular αS levels (Fig. 2c,f) was important for our subsequent analysis of fPD mutations: even if αS expression levels differ somewhat among mutants for biological or technical reasons, comparisons of the ratios should be meaningful.

The linear correlation of αS60 and αS14 levels across the expression range we tested suggests that these levels are theoretically in the same range as the apparent equilibrium constant. However, protein assembly systems in living cells are not in a true equilibrium but rather in a dynamic flux influenced by factors like biosynthetic and degradation rates and changes in subcellular localization. In this context, it is noteworthy that the αS concentrations we obtained by transient expression in human neuroblastoma cells (0.15–1.65 ng αS per μg of total cytosolic protein when using a range of 1–8 μg DNA transfected into 6-cm dishes) did not exceed the αS concentration in lysates of freshly biopsied human brain (1.8 ng μg^−1^), as quantified by αS enzyme-linked immunosorbent assay (ELISA) and confirmed by immunoblotting (Fig. 2g). Indeed, our measured concentrations are lower than the endogenous neuronal concentration in brain because brain lysate includes proteins from many non-neuronal cells having low αS. Overall, we achieved generally similar PBS-soluble (cytosolic) αS levels when we repeatedly expressed WT or five αS mutants in M17D cells (Fig. 2h). Despite a putative function in membrane vesicle homeostasis, αS has been reported to be predominantly cytosolic^13^,^24^,^25^, consistent with our data in primary neurons^9^ and human brain (Fig. 1a). In accord, our ELISA detected a very high portion (>85%) of cellular αS in the cytosol; the TX-100-soluble membrane fractions contained minor amounts (<15%) (Fig. 2i). Highly insoluble cellular fractions (that is, sequential 2% SDS and 88% formic acid extracts) contained very minor αS amounts, with no obvious effects of the 5 fPD mutations. Our ELISA was validated as detecting both normal and aggregated forms of αS (Supplementary Fig. 6). Western blots likewise detect only minor αS amounts in the 2% SDS-soluble fraction^9^. These relative distributions across fractions were true for both WT and fPD-mutant αS even after intact-cell crosslinking (Fig. 2i). Therefore, in our many iterative experiments below, we focused on the PBS-soluble (cytosol) major fraction but included some analyses of the TX-100-soluble minor fraction.

### fPD missense mutations lower the αS multimer:monomer ratio

**Figure 3:**
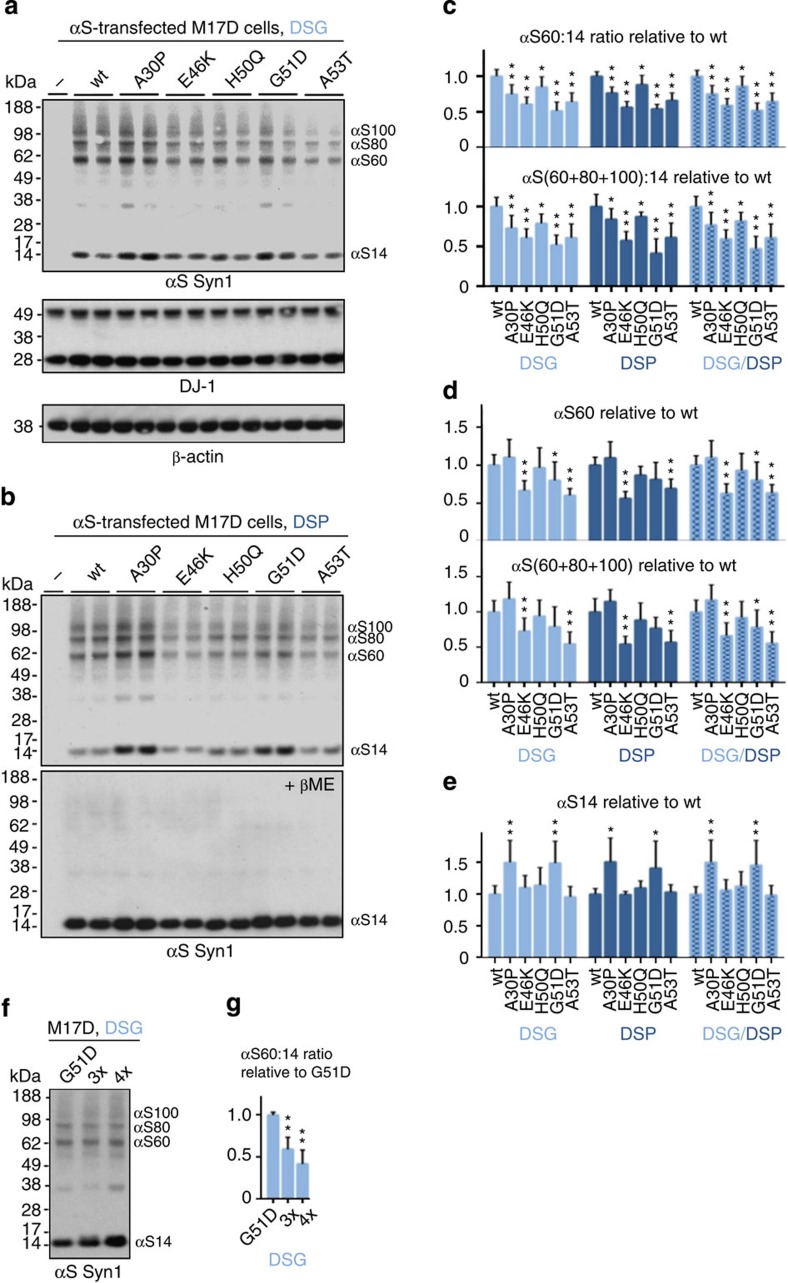
Intact-cell crosslinking of fPD-linked αS missense mutations. (**a**) DSG crosslinking analysis of M17D cells transiently transfected with αS WT or the indicated mutations. Western blots for endogenous DJ-1 and transfected αS in duplicate (Syn1); each lane is one transfection. (**b**) Analagous to Fig. 2a, but using the reducible crosslinker DSP: upper panel, non-reduced: bottom panel, βME-reduced (Syn1). (**c**) DSG and DSP crosslinking, plus meta-analysis of both: intensity of αS60 alone (upper panel) or αS60+80+100 (lower panel) is graphed relative to WT αS (set to 1) (DSG: *N*=8 experiments each done in biological duplicates (*n*=2) on different days, total *n*=16; DSP: *N*=4, *n*=9). For better visibility in this and the following graphs, only one-direction error bars are shown. (**d**) DSG and DSP crosslinking plus meta-analysis: levels of αS60 alone (upper panel) or αS60+80+100 together (lower) relative to WT αS; (**e**) level of αS14 alone relative to WT αS. (**f**) Representative western blots of DSG crosslinking of 3x (H50Q-G51D-A53T) and 4x (3x+E46K) compound fPD mutants relative to G51D alone. (**g**) Quantification of αS60:14 ratio for 3x and 4x compound fPD mutants relative to G51D alone (*N*=4, *n*=8). *P<0.05, **P<0.01; one-sided ANOVA (see Methods) for all quantifications shown; error bars, s.d.

To determine whether the fPD-causing αS mutations A30P, E46K, G51D and A53T (tested by fluorescence complementation in Fig. 1h) and the recently discovered H50Q^26^ alter the tetramer:monomer ratio in intact cells, we initially transfected human M17D neural cells with WT αS or one of these five fPD mutants and performed DSG crosslinking and quantitative western blots of the cytosols (Fig. 3a, top panel). Trapping of the endogenous DJ-1 dimer confirmed equal crosslinking across samples (Fig. 3a, middle panel; Supplementary Fig. 7 for uncropped blots). We identified Syn1 as the optimal antibody to detect αS because it is widely used and commercially available (unlike 15G7 or our 2F12), it does not show a preference for monomeric or multimeric αS (C20 reacts more with monomers, and 211 and LB509 do not detect multimers^9^), and it gave the clearest blots of the αS transfectants. Thus, statistical analyses of the experiments that follow used Syn1, but importantly, other αS antibodies confirmed all key findings in this section. In many iterative experiments, we detected similar or slightly reduced αS expression levels for the mutants versus WT, except for A30P and G51D, which showed variable but modestly higher αS levels (Fig. 3a,b; compare with Fig. 1h). These variations were well within the αS expression range found not to alter the αS60:14 ratio (see Fig. 2). Densitometry of multiple independent experiments after intact-cell crosslinking with DSG (exemplified by Fig. 3a) or DSP (exemplified by Fig. 3b) and a meta-analysis of the two agents revealed highly significant decreases (*P*<0.01, *n*=25, one-sided analysis of variance (ANOVA), percentages±s.d.; statistical details in Fig. 3 legend and Methods) in the αS60:14 ratio for A30P (77.5±15.5% of WT, that is, a 22.5% decrease), E46K (61.7±10.1% of WT, a 38.3% decrease), G51D (47.8±10.0% of WT, a 52.2% decrease) and A53T (66.5±10.4% of WT, a 33.5% decrease). H50Q caused a smaller (83.7±14.4% of WT, 16.3% decrease) but still significant (*P*<0.05) reduction (Fig. 3c, upper panel). The same significant effects were observed when the ratio of all multimeric species (αS60+80+100 combined) relative to αS14 was analysed (Fig. 3c, lower panel). We observed decreases versus WT in αS60 levels (Fig. 3d, upper panel) or αS60+80+100 levels (Fig. 3d, lower panel) for E46K, G51D and A53T, while the αS60 levels for A30P and H50Q were not significantly changed from WT. αS14 monomer levels increased significantly versus WT for A30P and G51D (Fig. 3e). Strikingly, engineered compound fPD mutants ‘3x' (H50Q+G51D+A53T) and ‘4x' (H50Q+G51D+A53T+E46K) further decreased the αS60:14 ratio compared with G51D alone, causing clear-cut elevations of free monomers (Fig. 3f,g). We then tested the overall finding—reduction of relative cellular multimer levels by fPD mutants—in a system that is closer to the steady-state situation in neurons, namely stable lentivirus transduction of the neural M17D cells with WT, E46K or G51D αS. The fPD mutants again reduced the cytosolic multimer:monomer ratio (due to both a decrease in multimers and increase in monomers versus WT), and sequential extractions again showed that non-cytosolic fractions contained only minor αS amounts (Supplementary Fig. 7).

### Decreased multimers in hA53T mice and A53T iPSC neurons

**Figure 4:**
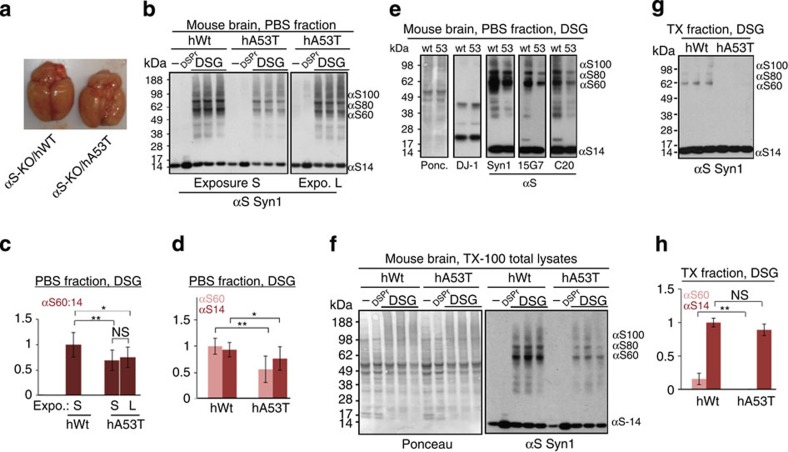
Transgenic hA53T versus hWT αS expressed in *αS-/*- mouse brain. (**a**) Whole brains from both genotypes immediately before mincing. (**b**) Minced brain bits from both mouse genotypes were subjected to crosslinking, and PBS-soluble (‘cytosolic') fractions blotted (Syn1). Untreated (-), DSP/βME-treated (DSPr) and DSG-treated samples (in technical triplicate) are shown at identical short (S) exposures of hWT and hA53T samples (left panel) or a longer (L) exposure of the hA53T samples (right panel: exposure matched to αS14 intensity of hWT αS in the left panel). (**c**) Densitometry of the cytosolic αS60:14 ratios (relative to hWT, set to 1) based on both S and L exposures (*N*=3 mice of each genotype analysed on different days in triplicates of separate brain-bit samples, total *n*=9); NS, not significant. (**d**) Densitometry of cytosolic αS60 and αS14 bands in both genotypes based on identical exposures (*N*=3, *n*=9); values relative to hWT αS60. (**e**) DSG crosslinked mouse brain samples: cytosols blotted for αS (Syn1, 15G7, C20) and DJ-1; Ponceau-staining of the blot membrane is on left. DJ-1 served as control for equal crosslinking efficiency and equal loading. (**f**) Minced brain bits from both genotypes: TX-100 total homogenates (cytosolic and membrane proteins). Untreated (-), DSP/βME-treated (DSPr) and DSG-treated samples in triplicates (Syn1 mAb, right panel). Left panel, Ponceau-staining of the membrane. (**g**) DSG crosslinking of PBS-insoluble TX-100-soluble (TX) fractions of brain from both genotypes in triplicates. (**h**) Densitometry of αS60 and αS14 in the TX fractions (*N*=2 mice of each genotype analysed on different days in triplicates of separate brain bits, total *n*=6); values relative to those of hWT αS14. *P<0.05, **P<0.01; Student's *t*-test (see Methods) for all quantifications shown; error bars, s.d.

**Figure 5:**
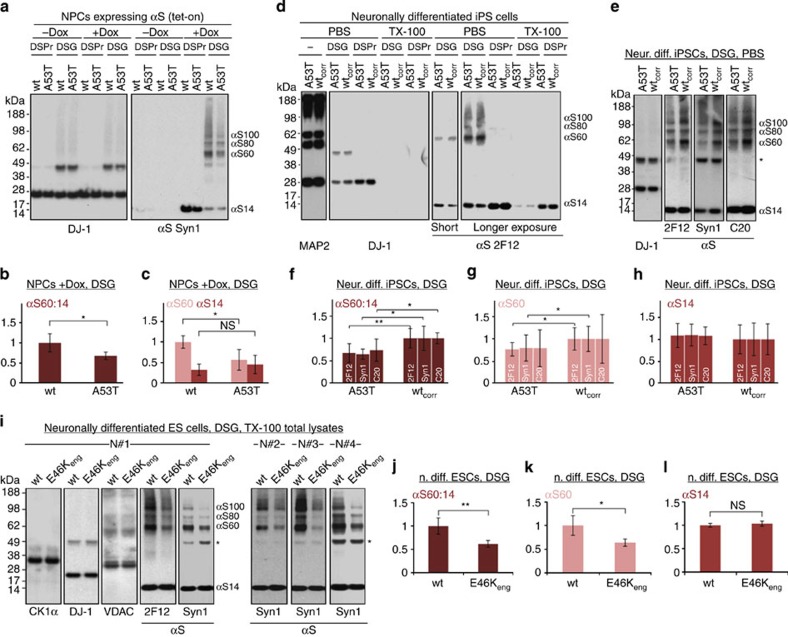
A53T versus WT αS in hESC- and hiPSC-derived neurons. (**a**) Crosslinking analysis of non-induced (-Dox) and induced (+Dox) hESC-derived NPCs expressing WT or A53T αS: PBS-soluble fraction. DSP/βME-treated (DSPr) and DSG-treated samples were blotted for DJ-1 and αS (Syn1). (**b**) Quantification of the cytosolic αS60:14 ratios (*N*=3 different cultures, each analysed in parallel in duplicates, total *n*=6); ratios relative to WT αS. (**c**) Quantification of cytosolic αS60 and αS14 for both genotypes (normalized to WT αS60); NS, not significant. (**d**) Crosslinking analysis of neurons differentiated from human A53T iPSCs versus their genetically corrected isogenic WT line (WT_corr_). PBS and TX-100 fractions of untreated (−), DSP/βME-treated and DSG-treated cells were probed for DJ-1 and αS (2F12). (**e**) DSG-crosslinked samples: cytosols blotted for αS (2F12, C20, Syn1) and DJ-1; * non-specific band detected only by Syn1 (ref. 9). (**f**) Densitometry of the cytosolic αS60:14 ratios (relative to WT) as detected by 2F12, C20 and Syn1 (*N*=4 cultures grown independently and analysed on different days in biological duplicates or triplicates; total *n*=10). (**g**) Densitometry of cytosolic αS60 (relative to WT) for both genotypes. (**h**) Densitometry of cytosolic αS14 (versus WT) for both genotypes. (**i**) Crosslinking analysis of neurons differentiated from WT hESCs or from a genetically engineered isogenic E46K line (E46K_eng_). TX-100 total protein lysates of DSG-treated cells were probed with casein kinase 1α (cytosolic monomer), DJ-1 (cytosolic dimer), VDAC (membrane-associated dimer) and αS (mAbs 2F12 and Syn1). The five panels on the left are from one experiment (N#1), the three panels on the right show the Syn1 western blots from three independent experiments (N#2-4, each quality-controlled by DJ-1 and VDAC western blots). *non-specific band detected only by Syn1 (ref. 9). (**j**) Densitometry of the cytosolic αS60:14 ratios (relative to WT) as detected by Syn1 mAb (N=4 cultures grown independently). (**k**) Densitometry of cytosolic αS60 (versus WT) for both genotypes. (**l**) Densitometry of cytosolic αS14 (versus WT) for both genotypes. *P<0.05, **P<0.01; Student's *t*-test (see Methods) for all quantifications shown; error bars, s.d.

Our method of crosslinking fresh, minced brain tissue (Fig. 1; Supplementary Fig. 1.; Methods) presented the opportunity to analyse the effects on αS tetramers of a PD-causing αS mutation expressed in mouse brain. We examined the brains (Fig. 4a) of young *αS-/*- mice expressing relatively low levels of human WT (hWT) or A53T (hA53T) αS^27^. Comparing total αS levels among samples using both untreated and DSP+β-mercaptoethanol (βME)-treated samples confirmed that DSP+βME facilitates the quantitative detection of total αS immunoreactivity on blots (Fig. 4b)^16^. Consistent with the brain expression data reported for these mouse lines^27^, PBS extracts (‘cytosol') of the hA53T brain showed somewhat lower total αS than the hWT brain (Fig. 4b). The αS60:14 ratio was significantly reduced (*P*<0.05, Student's *t*-test, *n*=9) in hA53T brain cytosols (Fig. 4c), due to a greater decrease in αS60 tetramer than αS14 monomer in hA53T versus hWT (Fig. 4d). To show that this ratio difference was not an artifact of the somewhat lower total αS levels in the hA53T brain, we included longer (L) exposures for hA53T that were adjusted to match the αS14 intensity of the hWT sample (Fig. 4b: ‘expo. L'). Densitometry on both exposures led to closely similar results of >20% reduction, namely, an αS60:14 ratio for hA53T versus hWT of 69.4±20.2% (‘S') or 75.3±20.1% (‘L'), (*P*<0.05, *n*=9, Student's *t*-test), with no significant difference in this result between the two exposures (Fig. 4c). This lower αS60:14 ratio in the hA53T mouse brain cytosol was consistently observed with Syn1 and confirmed with antibodies 15G7 and C20 (Fig. 4e). As expected, the hA53T αS60:14 ratio was also reduced when we analysed total lysates of whole mouse brain (that is, homogenizing the crosslinked tissue directly in TX-100) (Fig. 5f). In the membrane (TX-100) fractions, we found no significant reduction of αS14 monomer levels in hA53T versus hWT; as in the fresh human brain (Fig. 1a), tetramers/multimers were very low in abundance in this fraction and were almost undetectable for hA53T (Fig. 4g,h).

Given that the human A53T mutation corresponds to rodent WT αS at that codon, possibly masking pathological effects in the mouse brain, we sought to validate the A53T effect directly in a disease-relevant human cell model. To preclude experimental variation resulting from random genomic integration of a transgene, we initially used human embryonic stem cells (hESCs) that express human αS from a defined genetic ‘safe habour' locus under the control of a doxycyline (Dox) inducible promoter, using a zinc-finger nuclease (ZFN)-based gene editing strategy^28^,^29^ (Methods). These hESCs carrying either WT or A53T αS were differentiated into neural precursor cells (NPCs), which have low endogenous αS expression (Methods). Adding Dox for 4 days produced robust expression of the WT and A53T transgenes (Fig. 5a). These human NPCs closely reproduced our mouse brain observation: a ∼30% lower αS60:14 ratio for A53T than WT (67.9±9.1% of WT, *n*=6, *P*<0.05, Student's *t*-test; Fig. 5b), and a reduction in total A53T αS immunoreactivity (Fig. 5a) due to lower αS60 levels (Fig. 5c). Moreover, this NPC system indicated that neither long expression (>4 days) nor full neuronal differentiation is necessary for αS tetramer formation.

We then conducted quantitative analyses in neurons differentiated from an induced pluripotent stem cell (iPSC) line of a living PD patient harbouring the A53T mutation versus its corrected isogenic line (WT_corr_; ref. 30; Methods). Abundant microtubule-associated protein 2 (MAP2) expression in both lines indicated equal neuronal differentiation (Fig. 5d, left panel). Following intact-cell crosslinking and sequential extraction, the cytosols showed similar crosslinking efficiency based on DJ-1 dimer levels, and we again detected lower αS60:14 ratios for A53T than WT using three αS antibodies (Fig. 5d–f). Quantification of 3 independent experiments done on different days in biological duplicates or triplicates (*n*=10) confirmed a >25% significantly reduced αS60:14 ratio in the hA53T neurons using three different antibodies (66.9±21.0% of WT_corr_ for 2F12; 64.4±11.3% of WT_corr_ for Syn1; and 73.3±24.8% of WT_corr_ for C20; *P*<0.05 or <0.01 depending on antibody, Student's *t*-test; Fig. 5f). This was again due to a lower αS60 level (Fig. 5g,h), a finding entirely consistent across antibodies and experiments. In a similar way, we confirmed the effect of E46K (Fig. 5i), as initially determined in M17D cells (Fig. 3), in neurons derived from isogenic hESCs in which the E46K mutation had been inserted by genome editing^30^: the αS60:14 ratio was reduced by >35% (61.1±8.7% of WT, *P*<0.01, *n*=4, Student's *t*-test; Fig. 5j), mostly due to strongly reduced tetramer levels (Fig. 5k,l). Interestingly, the decrease in tetramers in these lines was closely similar to that seen in the earlier systems, even though these human neurons are heterozygous for A53T or E46K αS, suggesting a possible dominant-negative effect on tetramer formation.

### Amplifying E46K leads to less multimers and induces toxicity

**Figure 6:**
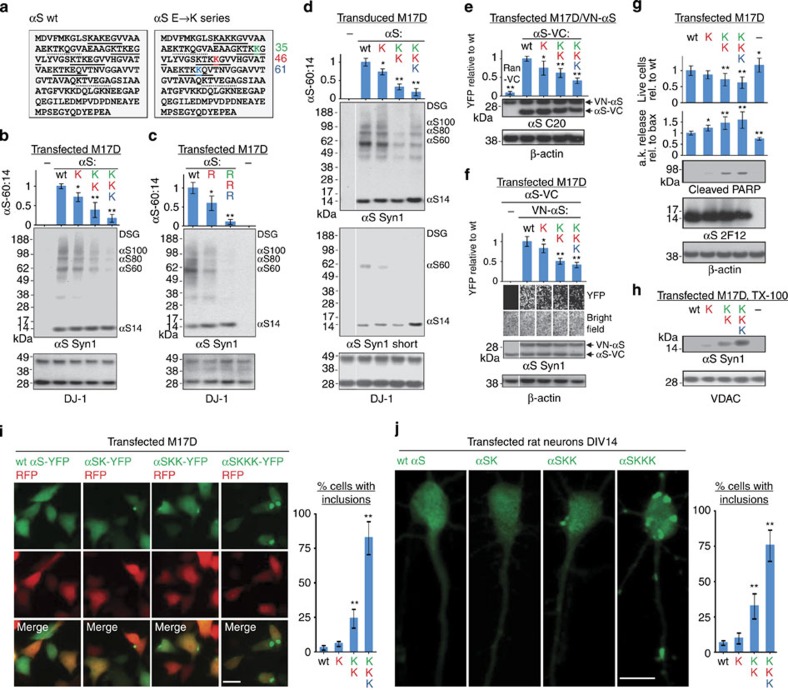
Multimers/toxicity/inclusions with 1-3 E46K-like mutations. (**a**) Schematic of human αS sequence showing engineered mutations (color-coded). Highly conserved KTKEGV motifs underlined, less conserved motifs dotted. (**b**) DSG-treated M17D cells expressing the indicated αS variants lysed in PBS/1% TX-100; graph (αS60:14 ratios by western blot densitometry) and western blots (Syn1, anti-DJ-1) represent *N*=3 experiments on different days. For this and the following graphs, WT set to 1 unless stated otherwise. (**c**) Analogous to (**b**) but 1 or 3 E→R substitutions. (**d**) Analogous to (**b**) using lentiviral pools; long and short western blot exposures, blots cut once as indicated. (**e**) Venus-YFP complementation assay by automated fluorescence reading. M17D/VN-αS cells transfected with αS-VC WT or mutant or Ran-VC (negative control). YFP fluorescence relative to WT; *N*=6 independent experiments on 3 different days using different DNA preparations. Representative western blots for αS (pAb C20) plus loading control β-actin. (**f**) Venus-YFP complementation assay by fluorescence microscopy; αS-VC (always WT) and 3 indicated VN-αS mutants were co-expressed (or not: -) in M17D cells; representative bright-field or fluorescent images and corresponding western blots (Syn1; β-actin as a loading control; blots cut as indicated). *N*=8 independent transfections on 3 different days. (**g**) Cytotoxicity assays: trypan blue exclusion for live cell count (*N*=18) relative to WT αS, Toxilight assay for adenylate kinase (a.k.) release relative to Bax (*N*=12), and western blot for cleaved PARP (representative of 6 independent experiments), each transfected as indicated or mock (-); plus western blots for αS (2F12) and β-actin (total lysates PBS/1% TX-100). (**h**) Western blots for αS (Syn1) and VDAC in TX-100-soluble fractions of M17D cells transfected as indicated. (**i**) Fluorescence microscopy of live M17D cells co-expressing RFP plus indicated αS-YFP variants; scale bar, 10 μm. Percentages of cells with inclusions were counted in a blinded fashion (right: *N*=3; 100 cells each). (**j**) Fluorescence microscopy of rat neurons (DIV14) transfected with indicated untagged αS variants; immunofluorescence with human-specific mAb 15G7; scale bar, 20 μm. Percentages of cells showing inclusions, blinded counting (right: *N*=3; 100 cells each). *P<0.05, **P<0.01; one-sided ANOVA (see Methods) for all quantifications shown; error bars, s.d.

Whereas A53T occurs in a unique stretch within αS, E46K is located within a canonical KTKEGV motif, which occurs at least six times in αS with slight variation (Fig. 6a), resembles motifs in apolipoprotein A1, and may facilitate interactions with lipids^31^. These facts suggested an intriguing opportunity to validate our central hypothesis: we reasoned that the multimer-destabilizing effect of E46K (Figs 1h and 3) might be amplified by analogous mutations in other KTKEGV motifs. We analysed αS constructs with one, two or three E46K-like substitutions, targeting the highly conserved KTKEGV motifs #3 and #5 flanking the fPD E46K-harbouring motif #4 (Fig. 6a). Intact-cell crosslinking of transfected M17D cells revealed a striking ‘dose-dependent' destabilization of αS60 by each additional E→K substitution, with the triple-K mutant almost abolishing cellular tetramers (Fig. 6b; Supplementary Fig. 9 for all uncropped blots in this section). To exclude an artificial effect of these lysine substitutions on the lysine-directed DSG crosslinking reaction itself, we expressed analogous arginine mutations (that is, 1R=E46R, and 3R), which also change the negative charge to positive but do not offer additional sites for our lysine-directed DSG crosslinker. Like E→K, the E→R substitutions in these three adjacent αS repeat motifs caused similar sharp reductions of the αS60:14 ratio (Fig. 6c). Importantly, the αS14 monomer remained well expressed for all constructs. We confirmed these findings in independent pools of lentivirus-transduced E→K M17D cells (Fig. 6d), obtaining closely similar decreases in the αS60:14 ratio despite some variability in total αS expression. The stepwise decrease in tetramer level was associated with a stepwise increase in monomer level, strongly supporting our overall hypothesis.

These consistent decreases in multimer formation documented by crosslinking offered an opportunity to independently quantify the E→K effect using our Venus-YFP complementation assay (Fig. 1g,h) and thus further cross-validate both methods. As a first step, we used the same protocol as in Fig. 1h by transfecting WT or E→K mutated αS-VC constructs into our VN-αS WT stable M17D line, followed by automated quantification of YFP-fluorescence. We observed a stepwise decrease in αS complementation with each additional E→K substitution, and with no consistent differences in total αS expression as detected by immunoblotting (Fig. 6e). Next, we used the opposite setup, introducing the E→K mutants into VN-αS constructs and co-expressing them in M17D cells with stable WT αS-VC. To quantify the resultant complementation, we chose areas from de-identified (‘blinded') cultures having closely similar cell densities by bright-field microscopy and examined many fields by fluorescence microscopy (Fig. 6f, upper panels). As always, stronger fluorescent signals (white) indicate greater complementation. There was a striking stepwise decrease of fluorescence with each additional E46K-like substitution in the repeat motifs (Fig. 6f). Immunoblotting with different αS and control antibodies excluded differences in protein levels (Fig. 6f, lower panels), and expressing αS-VC alone produced no fluorescence (Fig. 6f). In primary rat neurons, co-expression of αSKKK-VC and VN-αSKKK led to very low complementation that was virtually below detection limit, while both variants were clearly expressed, as shown by immunofluorescence using human αS-specific antibody 15G7 (Supplementary Fig. 10).

The analysis of single fPD mutants in acute cell culture has not produced consistent effects as regards their cytotoxicity^31^, in keeping with fPD being a very gradual disease of relatively late onset, but we reasoned that our ‘amplified' E46K tetramer-abrogating effect might produce measurable cytotoxicity in cultured neural cells. We thus expressed WT αS and all three E→K variants (1, 2 and 3K) in human M17D cells and observed a highly significant stepwise reduction in cell viability with each E→K substitution added to E46K, as documented by both trypan blue exclusion and an adenylate kinase release assay (Methods); the effect of E46K alone versus WT was noticeable but relatively weak (Fig. 6g, upper two panels). Importantly, these decreases in cell viability were accompanied by opposite increases in cleaved PARP, a widely used indicator for the activation of apoptosis (Fig. 6g, third panel), and again the single E46K alone had a weak but noticeable effect. These results were not due to differences in expression levels (Fig. 6g, fourth and fifth panels). When we asked whether this increase in neurotoxicity was paralleled by changes in αS solubility, we found increased total αS immunoreactivity in PBS-insoluble fractions such as the TX-100-soluble fraction (Fig. 6h) with each additional E→K substitution, starting with just 1K. Moreover, in live M17D cells, YFP-tagged αS-KKK showed a strong tendency to form round cytoplasmic inclusions (in 79±8.1% of cells; Fig. 6i), and this was weaker (23.5±5.9% of cells) but still highly significant (*P*<0.01) in αS-KK and less so but still noticeable (4.5±1.0% of cells; *P*>0.05) in αS-K versus αS-WT (2.3±0.6%), whereas the co-transfected soluble RFP control protein remained uniformly cytosolic (Fig. 6i). Importantly, we obtained highly similar results when we expressed untagged WT αS and the E→K mutants in primary rat neurons (DIV 12) and quantified the prevalence of cells harbouring discrete round inclusions in cell bodies and/or neurites (Fig. 6j).

## Discussion

αS accumulates in β-sheet-rich fibrils in numerous neurodegenerative diseases. For nearly two decades, αS was believed to exist normally as a natively unfolded, 14.5 kDa monomer^14^,^32^, and most papers on αS have emphasized this assumption in their introductions. In contrast, we recently reported that cellular αS can occur physiologically in partially α-helical, aggregation-resistant ∼60 kDa tetramers^7^ and that these may be in a complex equilibrium with monomers also normally present in neurons^9^. This unexpected finding has been controversial^13^,^14^. Here we document that αS exists as a ∼60 kDa tetramer in fresh, normal human brain tissue. Then, we show in multiple systems that all fPD-causing αS missense mutations significantly decrease tetramer:monomer ratios in neural cells, including in human neurons derived directly from a patient with the A53T mutation or WT human neurons genetically engineered to express E46K. This invariant effect by mutations known to unequivocally cause clinical PD strongly suggests that a tetramer-to-monomer destabilization facilitates subsequent pathological aggregation of monomers into disease-mediating assemblies. We validate this concept in Fig. 6: by inserting the PD-causing mutation at E46K into the analogous sites of first one and then two adjacent repeat motifs, we simultaneously link a stepwise decrease in tetramers/multimers and increase in monomers inside neurons (quantified by two methods) to a stepwise increase in neurotoxicity (quantified by three independent assays) and accumulation of αS in round cytoplasmic inclusions. This elucidation of a genotype-to-phenotype relationship for five fPD mutations, the initial pathogenic mechanisms of which have remained unclear, provides a compelling new model for how α-synucleinopathy can be initiated in PD and related disorders.

Each fPD-causing mutation reduced the intracellular αS60:14 ratio by 10–40% depending on the mutant, and engineered triple (H50Q-G51D-A53T) and quadruple (triple+E46K) fPD mutants produced even larger decreases, with sharp rises in free monomers. YFP complementation (analysed in a blinded fashion) showed highly significant effects of αS mutations in the same direction. We confirmed the fPD-mutant effect in the most relevant available systems: *αS-/*- mouse brain having low transgenic expression of human A53T αS, iPSC-derived neurons obtained from an A53T patient and engineered E46K hESC-derived neurons. This consistency across multiple cell/tissue sources and with two distinct techniques validates the mutational effects. The multimer-destabilizing effect and concurrent slight neurotoxicity of the E46K fPD mutation (Fig. 6g) was significantly 'amplified' by adding just one or two E46K-like mutations in the adjacent KTKEGV motifs. Our observation that intact six-residue repeats contribute to the normal αS tetrameric structure is of interest because analogous repeats occur in apolipoprotein A1 (ref. 31), which forms native low-*n* multimers in its lipid-free state^33^,^34^,^35^ and was crystallized as a tetramer^36^. Our multiple cellular and brain tissue experiments suggest that all of the clinical missense mutations destabilize the physiological tetramer, eventually leading to higher relative levels of unfolded monomer, the form believed to be the starting point for pathological αS aggregation^37^. Our results across the five fPD mutations showed a destabilization of tetramers most clearly, while the accumulation of monomers in our short-term experiments varied and achieved significance for A30P and G51D (Fig. 3e). Neurons are likely to compensate for unfolded αS monomers by degrading or clearing them in the short-term, but over longer periods or a human lifetime, this may not suffice to control the potential aggregation of excess monomers arising chronically from destabilized tetramers. In this context, we previously found that the native, helical tetramer purified from WT human erythrocytes showed no aggregation upon extended *in vitro* incubation, whereas unfolded monomers readily aggregated into β-sheet-rich fibrils (see Fig. 3d in ref. 7).

Two technical advances over our previous protocol for intact-cell crosslinking^9^ enabled these new findings. First, we developed a procedure to efficiently crosslink intracellular proteins in fresh brain samples that largely excludes broken cells. The latter step is key to studying αS because upon cell lysis, tetramers of αS or βS are depolymerized and can no longer be trapped quantitatively in their *in vivo* state^9^. Applying this new protocol to human brain immediately after biopsy confirmed the major observations of our intact-neuron crosslinking^9^: an abundant αS60 species (plus some αS80 and αS100 multimers) in the PBS-soluble fraction, with only minor and primarily monomeric αS in the membrane fraction. The fresh, normal human brain showed tetramers and monomers but essentially no intermediate dimer and trimer species, entirely distinct from what would be seen with artifactual (non-specific) crosslinking of the highly abundant αS protein, which in unfolded recombinant form produces a stochastic ladder of monomers>dimers>trimers>tetramers and so on. (see Fig. 4c in ref. 9). Of note, the known tetrameric protein p53 behaved similarly to αS in both our (Fig. 1d) and previous^18^ crosslinking assays: pronounced tetramers, few dimer or trimer intermediates, some apparent higher assemblies, and smears on overcrosslinking. And as before^9^, known monomeric proteins were not made into artifactual oligomers by our crosslinking (Fig. 1). Our new tissue protocol also revealed abundant αS tetramers in normal mouse brain, and the non-pathogenic homolog βS showed a closely similar pattern. Collectively, these results substantiate that the cellular tetramer detected by crosslinking is a physiological, not pathological or artifactual, assembly.

Second, by stringently standardizing our protocol to make it quantitative, we addressed novel questions relevant to PD pathogenesis: are cellular tetramer:monomer ratios affected by αS expression levels and by PD-causing αS mutations? We generated data indicating that (a) the cellular tetramer:monomer ratio is constant over a relatively wide range of αS expression levels that were similar to but always somewhat lower than those in human brain tissue; and (b) mutations which cause fPD shift the ratio away from the tetramer. The steady rise in tetramer levels with increasing αS expression (Fig. 2) suggests that physiological tetramerization is either an intrinsic feature of the αS polypeptide chain or any transacting co-factors needed for tetramer assembly and stabilization (for example, membrane vesicles, free lipids and so on) are not limiting in our cells at these αS expression levels.

Two recent studies confirm the existence of physiological multimers (including tetramers) in neurons^21^,^38^, and one of these^21^ provides functional evidence that a portion of αS multimers can localize to synaptic vesicles, cluster them and prevent their exocytosis, which could downregulate neurotransmitter release. Nonetheless, data from our and several other^7^,^8^,^15^,^39^ labs suggest that native αS multimers are mainly present in the cytosol. Westphal and Chandra^15^ confirmed the purification of α-helical αS assemblies from erythrocytes using our methods and underscored their soluble nature. A study on γ-synuclein suggested that this αS homolog is tetrameric unless it binds to heterologous binding partners or membranes^40^, in agreement with our finding that cytosolic but not membrane-associated αS molecules are recovered as multimers. Wang *et al*.^8^ reported NMR analyses of an N-terminally modified αS construct expressed in *E. coli* and purified under non-denaturing conditions that formed α-helical tetramers. Their CD spectroscopy of WT and fPD recombinant proteins revealed decreasing α-helical content in the order WT>A30P>A53T>E46K, closely resembling the relative order of the αS60:14 ratios in our assays (see Supplementary Fig. 11). The reported ages of symptom onset in humans carrying fPD missense mutations may agree in general with the relative decrease in our αS60:14 ratios. G51D causes very early onset PD similar to αS triplication^41^,^42^, while symptoms from H50Q and A30P begin somewhat later^3^,^26^,^43^; we find the greatest lowering of the tetramer:monomer ratio for G51D and the least for A30P and H50Q (Fig. 3).

Tetramers and monomers are likely to be in a complex dynamic equilibrium in living cells, as proposed for p53 (refs 10, 11). The αS60 species (which we confirmed here by mass spectrometry to contain only αS on purification (Supplementary Fig. S3)) was representative of the αS80 and αS100 species (Supplementary Fig. S2), which may be conformers of the tetramer (discussed in ref. 9). When all αS-immunoreactive mid-MW bands (60, 80 and 100 kDa) are considered together, the multimer content of cultured cells and brain substantially exceeds the monomer content (2–3:1), as judged by antibodies that react similarly with both. And if some of the αS14 we observe represents ‘false-positive' monomers present because the crosslinking is not fully efficient, then the multimer:monomer ratio *in vivo* is even higher than 3:1. Whatever the absolute multimer:monomer ratios are *in vivo*, the consistent detection of multimers of endogenous αS and βS in normal cells by both *in situ* crosslinking and fluorescence complementation contrasts sharply with the long-held view that cellular αS assemblies above monomer are exclusively pathological.

How can our findings be synthesized into an integrated model for the conversion of physiological αS into pathogenic forms? The field has long accepted that contact between the ‘natively unfolded monomer' and negatively charged lipid vesicles *in vitro* induces α-helical structure^44^. If this finding has any *in vivo* relevance, we hypothesize that after their synthesis on the ribosome, unfolded αS monomers undergo a helical conversion and multimeric assembly, perhaps by transient contact with lipid vesicles followed by rapid detatchment, thus ending up principally in the cytosol, where we recover the tetramers quantitatively after *in vivo* crosslinking. Alternatively, an unknown small ligand, for example, a free lipid, could induce and/or maintain the folding and assemby state. Our inability to detect by mass spectrometry any other proteins as a component of the gel-purfied αS60 tetramer does not exclude the presence of one or more lipids in the assembly. Our findings (sequential extractions that leave αS60 principally in the cytosol (Fig. 1a); diffuse cytosolic signal of the multimers by YFP complementation (Fig. 1e–g)) argue against a persistent close contact between membranes and most tetramers/multimers at steady state.

Pathological polymerization may occur when the storage capacity of the cell's native tetrameric population is somehow exceeded, for example, destabilization by missense mutations leading to relatively more monomers, or else chronic elevation of all forms of the protein (for example, increased gene dosage^5^), leading to absolutely more monomers. In sporadic PD, different neuronal stressors could, over time, destabilize a small fraction of the WT tetrameric population to monomers, for example, through post-translational modifications such as phoshorylation, dopamine induced conformational changes, oxidative stress/free radical accumulation with age, chronic mitochondrial dysfunction, altered chaperone systems, metal ion dyshomeostasis, or extraneuronal amyloid-β accumulation (in Alzheimer's disease). In such pathogenic scenarios, which could underlie cases of ‘idiopathic' synucleinopathies, pathological αS aggregates would represent a secondary effect, whereas in αS mutation carriers, they would be considered primary.

The drastic alteration in αS multimerization state on cell lysis^9^ makes addressing its function challenging. αS cannot be easily studied in its native state outside of cells by conventional biochemical approaches because of the apparent dynamic flux between multimers and monomers that exists in intact cells^9^. Only techniques that reveal the intracellular state of αS and are also sufficiently sensitive to quantify the range of endogenous assembly forms should be used. At present, this means relying on quantifiable *in vivo* crosslinking with cell-penetrant agents that can trap αS and βS in their native multimeric states, and imaging of intact cells expressing tagged forms, although the latter approach has the twin disadvantages of modifying the αS structure and not distinguishing different-sized multimers. We have reported clear evidence that multi-step purification of endogenous αS from human cells and brain allows a portion of the purified protein to remain tetrameric during the native purification^7^,^45^.

The central therapeutic implication of our work is similar to that achieved for transthyretin^46^, a physiological tetramer stabilized by a natural ligand (thyroxin), destabilized by amyloidogenic missense mutations, and therapeutically stabilized by certain small molecules (for example, tafamadis). Similar strategies are being discussed for the cancer-relevant physiological tetramer of p53 (refs 47, 48, 49). The stabilization by small, brain-penetrant molecules of the αS tetramer is now a rational and attractive goal.

## Methods

All materials mentioned were purchased from Invitrogen unless stated otherwise.

### Brain samples

The experimental use of discarded human brain samples was approved under protocol number 1999P001180 ('Aging in the Brain: Role of the Fibrous Proteins') by the Partners Human Research Committee, the Institutional Review Board of Partners Research Management. Informed consent for the use of brain tissue was obtained from the subject undergoing brain biopsy. Rodent samples were acquired under protocol number 05022 (‘Mouse Models for Parkinson's Disease'), approved by the appropriate IACUC, the Harvard Medical Area Standing Committee on Animals. Wt mice (C57BL/6; 12 wk old) were from Charles River, Wilmington, MA, and transgenic mice (ref. 27; age-matched pairs, male, 6–9-week old) from Jackson labs, Bar Harbor, ME. The human cerebral cortex biopsy was obtained fresh from a patient undergoing focal ablative surgery for epilepsy who was otherwise healthy and had no synucleinopathy or other neurodegenerative disease; the sample was obtained from unaffected, discarded tissue and analysed fresh (storage on ice about 2 h).

### Cell culture and transfection

Cells were cultured at 37 °C in 5% CO_2_. Human erythroid leukemia cells (HEL; ATCC number TIB-180) were cultured in RPMI 1640 (ATCC modification) supplemented with 10% fetal bovine serum (Sigma), 50 units per ml penicillin, and 50 μg ml^-1^ streptomycin. Human neuroblastoma cells (BE(2)-M17, called M17D; ATCC number CRL-2267) were cultured in DMEM supplemented with 10% fetal bovine serum (FBS), 50 units per ml penicillin, 50 μg ml^−1^ streptomycin, and 2 mM L-glutamine. M17D cells were transfected using Lipofectamine 2000 according to manufacturer's directions. Cells were normally collected 48 h after transfection. Primary neurons were cultured from E18 Sprague-Dawley rats (Charles River, Wilmington, MA). Rats were euthanized with CO_2_ followed by cervical dislocation. Embryonic cortices were isolated and dissociated with trypsin and trituration. Cells were plated in DMEM supplemented with 10% FBS, 50 units per ml penicillin, 50 μg ml^-1^ streptomycin, and 2 mM glutamine at 680 cells per mm^2^ on BioCoat poly-D-lysine-coated culture dishes (BD Biosciences). After 4 h, medium was changed to Neurobasal medium supplemented with B-27, 2 mM GlutaMAX, and 50 μg ml^-1^ gentamicin. Half of the medium was replaced every 4 days; on the first medium change, 5-fluoro-2-deoxyuridine (Sigma) and uridine (Sigma) were added to concentrations of 100 and 500 μg l^-1^, respectively.

### cDNA cloning

Single-mutation αS expression plasmids were generated from the pcDNA4/αS plasmid^9^ using the QuikChange II site-directed mutagenesis kit and appropriate primers. Multiple-mutation constructs were synthesized as GeneArt Strings DNA fragments (GeneArt/Life Technologies), and inserted into pcDNA4 with the In-Fusion HD Cloning Kit (Clontech). pcDNA3/VN-αS and pcDNA3/αS-VC WT and fPD mutant constructs were gifts of T.F. Outeiro, Goettingen). VN and VC constructs for insertion into pcDNA4/TO/myc-his A (pcDNA4) were synthesized by GeneArt, excised directly from the vector in which the constructs were delivered, and ligated into pcDNA4. αS tagged constructs were created by linearizing the resulting construct in pcDNA4 and inserting αS cDNA by In-Fusion. Full-length YFP was reconstituted from the VN and VC tags and inserting into pcDNA4; full-length tagged constructs were subsequently made by insertion of cDNA upstream of the tag. Lentiviral constructs pLenti6/αS WT, G51D, E46K, 2K and 3K were generated by In-Fusion with the pLenti6 vector and respective cDNA templates. Lentiviral construct pWPXL/αS-WT was generated by In-Fusion from pWPXL/eGFP (pW), a gift of Didier Trono (Addgene plasmid # 12257), without removing eGFP. pCAX/dsRed and pcDNA3.1/Bax were gifts from T. Young-Pearse and M. LaVoie, respectively. The following primers were used. To reconstitute full-length YFP and infused into pcDNA4: VN-to-FP_FW 5′- AGTGTGGTGGAATTCTGCAGATGGTGAGCAAGGGCGAGG -3′, VNVC-combine_FW 5′- CTATATCACCGCCGACAAGCAGAAGAACGGCATCAAGGCC -3′, VNVC-to-YFP_Rev 5′- GCCCTCTAGACTCGAGTTACTTGTACAGCTCGTCCATG -3′. To insert αS into pW (digested with BamHI and MluI): pW-αS-infu-BamHI-MluI_FW 5′- TTAAACTACGGGATCCATGGATGTATTCATGAAAGGA -3′ pW-αS-infu-BamHI-MluI_Rev 5′- TAGCGCTAGGACGCGTTAGGCTTCAGGTTCGTAGTC -3′. To insert αS variants into pcDNA4/VN-αS: αS-VN-FW_Infu 5′- TGGAGGTGGTGGATCCCTTAAGGATGTATTCATGAAAG -3′, αS-VN-rev_Infu 5′- GCGGCCGCCGATATCTTAGGCTTCAGGTTCGTAG -3′. To insert αS into pcDNA4/VC: αS-VC-fw_Infu 5′- AGTGTGGTGGAATTCTGCAGATGGATGTATTCATGAAAGG -3′, αS-VC-rev_Infu 5′- TGCCGTTCTTCTCGAGGGCTTCAGGTTCGTAGTC . To insert αS into pcDNA4/YFP p4-BamHI-αS-Infu_FW 5′- TACCGAGCTCGGATCCATGGATGTATTCATGAAAGGA -3′, p4-αS-PstI-FP-fus_Rev 5′- CCCTTGCTCACCATCTGCAGGGCTTCAGGTTCGTAGTC -3′. To insert Ran into pcDNA4/VC: p4-Ran-VC-Infu_FW 5′- AGTGTGGTGGAATTCTGCAGATGGCTGCGCAGGGAGAG -3′, p4-Ran-VC-Infu_Rev 5′- TGCCGTTCTTCTCGAGCAGGTCATCATCCTCATCC -3′. To insert αS WT, E46, KK and KKK into pLenti6: pLenti6-αS_FW 5′- GGATCCACTAGTATGGATGTATTCATGAAAGG -3′, pLenti6-αS_Rev 5′- GCCCTCTAGACTCGAGTTAGGCTTCAGGTTCGTAG -3′.

### Stable cell pools and cell lines

Lentiviral particles derived from pLenti6 were generated as recommended by the manufacturer. Lentivral particles derived from pWPXL were generated with plasmids psPAX2 and pMD2.G (also gifts from D. Trono. Addgene plasmids #12260 and 12259, respectively). M17D cells were transduced with viral particles according to the respective recommended protocols. The stable monoclonal cell line M17D/VN-αS was generated by transfection of M17D cells with pcDNA3/VN-αS, followed by G418 selection and isolation of single clones. Stable pools of pLenti6 transduced cells were generated by blasticidin selection, and stable pools of pWPXL transduced cells were selected for residual GFP expression with FACS.

### Human ES cell and iPSC cultures

hiPSCs and the hESC lines WIBR3 (Whitehead Institute Center for Human Stem Cell Research, Cambridge, MA) and BG01 (NIH code: BG01; BresaGen, Inc., Athens, GA) were maintained on mitomycin C-inactivated mouse embryonic fibroblast feeder layers in hESC medium (DMEM/F12 supplemented with 15% FBS (Hyclone), 5% KnockOut Serum Replacement, 1 mM glutamine, 1% nonessential amino acids, 0.1 mM β-mercaptoethanol (Sigma) and 4 ng ml^−1^ FGF2 (R&D systems))^30^. Cultures were passaged every 5-7 days either by trituration or enzymatically with collagenase type IV (Invitrogen; 1.5 mg ml^−1^).

### Gene-edited pluripotent stem cell lines

hESCs lines expressing human aS (WT or hA53T mutant) under the control of Dox from a defined genetic ‘safe harbor' locus were generated by a previously described ZFN-based genome-editing strategy. ZFNs were linked to WT FokI which was linked to an obligate heterodimer form of the Fok1 endonuclease (ELD-KKR)^28^,^29^. hESCs were simultaneously targeted in both alleles of the AAVS1 locus with two distinct donor plasmids, each containing a distinct selection marker (neomycin or puromycin resistance gene) followed by either a CAGGS promoter driving constitutively expressed reverse tetracycline transactivator (M2rtTA) or the αS transgene (WT or A53T) driven by a Dox-responsive element. Correctly targeted hESC clones derived from individual cells with unique integration of each donor plasmid in one of the AAVS1 alleles and lack of additional random donor integrations were identified by Southern blotting as described^28^. αS expression in NPC was induced by supplementing the medium with Dox at a final concentration of 2 μg ml^−1^. The A53T iPSCs plus genetically corrected isogenic line (WT_corr_) as well as the genetically engineered E46K hESC line plus parental WT αS hESC line had been generated before by the ZFN-based genome-editing strategy, as published in detail^30^.

### NPC culture and terminal differentiation

Differentiation into NPCs and then terminally differentiated neurons was according to reported protocols with slight modifications^28^,^50^. Briefly, hESC/hiPSC colonies were collected using 1.5 mg ml^−1^ collagenase type IV, separated from mouse embryonic fibroblast feeder cells by gravity, gently triturated and cultured for 8 days in non-adherent suspension dishes (Corning) in EB medium (DMEM with 20% KnockOut Serum Replacement, 0.5 mM glutamine, 1% nonessential amino acids, 0.1 mM β-ME (Sigma)) supplemented with 50 ng ml^−1^ human recombinant Noggin (Peprotech) and 500 nM dorsomorphin (Stemgent). Subsequently, human EBs were plated onto dishes coated with poly-L-ornithine (15 μg ml^−1^, Sigma), laminin (Sigma) and fibronectin (Sigma) in N2 medium supplemented with 50 ng ml^−1^ human recombinant Noggin, 500 nM dorsomorphin and FGF2 (20 ng ml^−1^, R&D Systems). After 8 days, neural rosette-bearing embryonic bodies (EBs) were microdissected, dissociated with 0.05% trypsin/EDTA solution and expanded on poly-L-ornithine, laminin and fibronectin coated dishes at a density of 5 × 10^5^ cells per cm^2^ in N2 medium supplemented with FGF2 (20 ng ml^−1^). Proliferating NPCs were passaged 2–3 times before induction of terminal differentiation into neurons by growth factor withdrawal in N2 medium supplemented with ascorbic acid (Sigma). Differentiated neurons were used for chemical crosslinking 45–55 days after induction of terminal differentiation.

### Immunocytochemistry

Cells were grown on poly-D-lysine-coated surfaces or coverslips, rinsed twice with HBSS with divalent cations, fixed 25 min at RT with 4% paraformaldehyde/PBS, then washed three times for 5 min with PBS^51^. Cells were then blocked and permeabilized with 5% BSA/0.25% Triton X-100/PBS. Cells were incubated with primary antibody in block-permeabilizing buffer for 2 h at RT or overnight at 4 °C. After incubation with primary antibody, cells were washed 3 × for 5 min with PBS, then incubated 1–2 h at RT with Alexa Fluor 488- and Alexa Fluor 568-coupled secondary antibodies diluted 1:2,000 in 5% BSA/PBS (no Triton). Cells were washed 3 × 10 min at RT with PBS, then analysed directly in the dish (Fig. 6j) or after mounting with Mowiol (Sigma) and coverslips (Fig. 1e,f).

### Intact-cell crosslinking of cultured cells

Crosslinkers were stored at 4 °C with desiccant. Cells were collected by trituration (HEL and M17D) or scraping (primary neurons), washed with PBS, and resuspended in PBS with Complete Protease Inhibitor, EDTA-free (Roche Applied Science). Crosslinkers were prepared at 50 × final concentration in DMSO immediately before use. Samples were incubated with crosslinker for 30 min at 37 °C with rotation. The reaction was quenched by adding Tris, pH 7.6, at 50 mM final concentration and incubated for 15 min at RT. After quenching, proteins were extracted (see below). Before crosslinking, one representative aliquot of the cells (for example, one extra 6-cm dish cultured for this purpose) was lysed in a volume of PBS/PI that was estimated to generate a protein concentration in the cytosol fraction of 1.5 μg ml^−1^ (lysis by sonication; 20,000 *g* spin, 30 min, 4 °C to collect cytosol). Then, the actual protein concentration was determined by BCA assay. On the basis of this result, the final volume of crosslinking buffer (PBS/PI+1 mM DSG or 1.75 mM DSP) for all the cell samples was adjusted so as to guarantee a 1.5 μg ml^−1^ cytosolic protein concentration in every sample at time of crosslinking. In general, samples with a measured concentration of <1.3 or >1.7 μg μl^−1^ were excluded from analyses. As an alternative, samples (for example, cells from one 10-cm dish) were split into different tubes of 1 mM DSG at different volumes (for example, 300, 400, 500 μL), followed by protein extraction, BCA assay and matching of samples that have similar protein concentration as an indication of similar protein-to-crosslinker ratios. The most important criterion for data inclusion was equal crosslinking observed for the DJ-1 control blots (no apparent differences in DJ-1 dimer:monomer ratios was a pre-established criterion^9^). Before crosslinking and further processing, sample order was randomized.

### Intact-cell crosslinking of fresh brain tissue

For intact-cell crosslinking of fresh brain, samples (mouse whole brain, human cortical biopsy) were finely minced by two rounds on a McIlwain Tissue Chopper (model MTC/2E, Mickle Laboratory Engineering Co., Gomshall, UK; blade interval: 100 μm); the sample was turned 90° after the first round. Minced samples were transferred to 15 ml tubes containing 5 ml PBS/PI, and the solution was resuspended by gentle shaking. Aliquots were transferred to 1.5 ml tubes and spun at 1,500 *g* for 5 min at RT. Supernatants were discarded, and the pellets (intact tissue bits) underwent crosslinking routinely at a ratio of 1 ml 1 mM DSG per 100 mg tissue or 1 ml 1.75 mM DSP per 100 mg tissue, while for DSG gradients we used volumes of 0.4–2 ml of 1 mM DSG per 100 mg tissue. The tissue suspensions were incubated at 37 °C for 30 min with shaking, followed by spinning at 1,500 *g* for 5 min at RT. After this intact-cell crosslinking, the supernatant was discarded, and the brain bits in the pellet were resuspended in PBS/PI (in 0.25 ml per 100 mg tissue), followed by protein extraction as described below.

### Protein extraction

Cells were lysed by sonication (Sonic Dismembrator model 300; microtip setting=40; 2 × 15 s). Spinning at 800 *g* for 5 min at 4 °C yielded the postnuclear supernatant (step was occasionally omitted), and this was centrifuged at 20,000 *g* for 30 min or ultracentrifuged at 100,000 *g* for 60 min at 4 °C to collect the supernatant (‘cytosol') and pellet (membranes). The latter pellet was resolubilized by sonication (2 × 15 s, amplitude 40) in PBS/1% TX-100 (same volume as cytosols), followed by centrifugation at 20,000 *g* for 30 min or ultracentrifugation at 100,000 *g* for 60 min at 4 °C. The resultant supernatant contained extracted membrane proteins; in some experiments, we added a 1 h 2% sarkosyl incubation of the TX-100-insoluble or a 4 h incubation of the LDS-insoluble pellet in 88% formic acid with shaking.

### Immunoblotting

Protein concentrations were determined by BCA assay (Thermo Scientific) according to the manufacturer's directions. Samples were prepared for electrophoresis by the addition of NuPAGE LDS sample buffer and boiling for 10 min. If not stated otherwise, 30 μg of total protein were loaded per lane. Samples were electrophoresed on NuPAGE 4–12% Bis-Tris gels with NuPAGE MES-SDS running buffer and the SeeBlue Plus2 MW marker. After electrophoresis, gels were electroblotted onto Immobilon-Psq 0.2 μm PVDF membrane (Millipore) for 1 h at 400 mA constant current at 4 °C in 25 mM Tris, 192 mM glycine, 20% methanol transfer buffer. After transfer, membranes were incubated in 0.4% paraformaldehyde, PBS for 30 min at RT, rinsed twice with PBS, stained with 0.1% Ponceau S in 5% acetic acid, rinsed with water, and blocked in 0.2% IBlock solution (PBS containing 0.1% (v/v) Tween 20 (PBS-T) and 0.2% (w/v) IBlock) for either 30 min at RT or overnight at 4 °C. After blocking, membranes were incubated in primary antibody in 0.2% IBlock with 0.02% sodium azide for either 1 h at RT or overnight at 4 °C. Membranes were washed 3 × 10 min in PBS-T at RT and incubated (45 min at RT) in horseradish peroxidase-conjugated secondary antibody (GE Healthcare) diluted 1:10,000 in 0.2% IBlock solution. Membranes were then washed 3 × 10 min in PBS-T and developed with ECL Prime (GE Healthcare-Amersham Biosciences) or SuperSignal West Dura (Thermo Scientific) according to manufacturers' instructions.

### Antibodies

Antibodies used were monoclonals Syn1 to αS (Clone 42, Becton-Dickinson; 1:3,000 in western blotting for transfectants, 1:400 in western blotting for endogenous αS), 2F12 to αS^9^ (0.09 μg ml^−1^ for transfectants and 0.9 μg per ml for endogenous in western blotting; 9 μg ml^−1^ in immunocytochemistry), SOY1 to αS (used in ELISA, see below for details), 15G7 to αS^52^ (hybridoma supernatants were 1:500 in western blotting for transfectants, 1:50 for endogenous αS, 1:5 in ICC), 71.1 to GAPDH (Sigma; 1:5,000 in western blotting), DLP1 to DRP-1 (Becton-Dickinson; 1:1,000 in western blotting), sc-126 to p53 (Santa Cruz, 1:1,000 in western blotting), EP1537Y to βS (Novus Biologicals, 1:3,000 in western blotting) and PRK8 to Parkin (Santa Cruz, 1:1,000 in western blotting), as well as polyclonal antibodies C20 to αS (Santa Cruz; 1:1,000 for endogenous αS and 1:5,000-1:10,000 for transfectants in western blotting), ab8227 to β-actin (Abcam; 1:5,000 in western blotting), ab5392 to MAP2 (Abcam; 1:1,000 in western blotting), PA1-954A to VDAC (Affinity Bioreagents; 1:1,000 in western blotting), D64E10 to cleaved PARP (Asp214) (Cell Signaling; 1:1,000 in western blotting), sc-126 anti-synaptobrevin-2 (Synaptic Systems, Göttingen, Germany; 1:1,000 in western blotting), anti-Ran (4462, Cell Signaling; 1:1,000 in western blotting), anti-casein kinase 1α (Sc-6477, Santa Cruz; 1:2,000 in western blotting), anti-DJ-1 (ref. 53, 1:3,000 in western blotting) and anti-GFP (T-19, Santa Cruz, 1:500 in ICC). 2F12 and SOY1 were generated by immunizing *αS-/*- (KO) mice with native αS purified from human erythrocytes. Hybridoma cell lines were generated by fusion of mouse splenocytic B lymphocytes with X63-Ag8.653 myeloma cells. Antibodies were purified from hybridoma supernatant by Cell Essentials (Boston, MA).

### αS-specific ELISA

Multi-Array High Bind plates (96-well plates; MSD, Meso Scale Discovery, Rockville, MD) were coated with the capture antibody 2F12 diluted (6.7 ng μl^−1^) in Tris-buffered saline with 0.05% Tween-20 (TBS-T) in 30 μL per well and incubated at 4 °C overnight. Liquid was removed and plates were blocked for 1 h at RT in blocking buffer (5% MSD Blocker A; TBS-T). After 3 washes with TBS-T, samples diluted in TBS-T with 1% MSD Blocker A and 0.5% NP40 were incubated at 4 °C overnight. Sulfo-tagged SOY1 mAb (for detection) was generated using Sulfo-Tag-NHS-Ester (MSD), diluted in blocking buffer (6.7 ng μl^−1^), added to the plate (30 μl per well) and shaken for 1 h at RT. After three washes, MSD Reader buffer was added, and plates were immediately measured using a MSD Sector 2400 imager.

### Densitometry and statistical analyses

Scanned western blots were analysed with the ImageJ software, version 1.47 (ref. 54). Pictures were inverted and usually the background signal from an empty lane was subtracted to obtain specific signals for each lane. *R*^2^ values (Fig. 2 and S4) were calculated using Microsoft Excel. WT αS was normalized to 1 in each independent experiment (*N*) to allow comparison between experiments done on different days. For statistical analyses of WT versus A53T (Figs 4, 5), we performed Student's *t*-test (tails=2, type=2; Microsoft Excel). For all other statistical analyses (Figs 1, 3), one-way ANOVA was routinely performed to determine significance versus WT αS using GraphPad Prism Version 6.05 under the program's guidelines (Tukey's multiple comparison's test, calculation of adjusted *P*-values, ‘repeated measures' correction where applicable). Where necessary, standard deviations for controls were obtained by calculating the deviation of duplicates or triplicates from their mean in each independent experiment. Normal distribution and similar variances were observed for both WT and mutant αS values. Graphs are means +/− s.d. Criteria for significance: *P*<0.05, *P<0.01, **. Sufficient experiments and replicates were run to achieve statistical significance.

### Cytotoxicity assays

The ToxiLight Non-destructive Cytotoxicity BioAssay Kit (Lonza) was used according to directions to measure adenylate kinase release; signals from untransfected cells were subtracted for normalization. For trypan blue assays, cell suspensions were mixed 1:1 with 0.4% Trypan Blue (Sigma) and counted on a TC10 automated cell counter (Bio-Rad).

### YFP complementation and microscopy

The principle of the YFP complementation assay, including controls for the specificity of the VN-αS/αS-VC interaction, has been described^22^,^23^,^55^,^56^. We routinely used a stable cell line M17D/VN-αS into which we transfected VC-tagged plasmids (1 μg DNA per well of a 24-well dish), followed by automated fluorescence detection (excitation 505 nM, emission 535 nm; Synergy H1 HybridReader, BioTek) 40 h post transfection. Alternatively, we co-expressed a constant WT αS-VC plasmid (4 μg per 6-cm dish) and a variable VN-αS plasmid (WT or point mutants; 2 μg per 6-cm dish) for 40 h in M17D cells followed by bright-field and fluorescence microscopy of live cells in culture dishes (AxioVert 200 microscope; AxioCam MRm camera; AxioVision Release 4.8.2; all by Zeiss, Jena, Germany). Images of YFP were collected using a GFP/FITC filter cube and are pseudo-colored green. GFP detection was enhanced by using an anti-GFP antibody. Microscopy analyses were performed in a blinded fashion by assigning random numbers to culture dishes by one investigator before representative images for each culture were taken or cells (+/− inclusions) were counted by another investigator. Confocal images were obtained on a Zeiss LSM710 system.

## Additional information

**How to cite this article**: Dettmer, U. *et al*. Parkinson-causing α-synuclein missense mutations shift native tetramers to monomers as a mechanism for disease initiation. *Nat. Commun.* 6:7314 doi: 10.1038/ncomms8314 (2015).

## Supplementary Material

Supplementary Information Supplementary Figures 1-11
