# Supplementary material for: Parkinson-causing α-synuclein missense mutations shift native tetramers to monomers as a mechanism for disease initiation
Source: Nat Commun. 2015 Jun 16;6:7314. doi: 10.1038/ncomms8314 (PMC4490410; doi:10.1038/ncomms8314)
Supplement: Supplementary Information — Supplementary Figures 1-11 [file ncomms8314-s1.pdf]

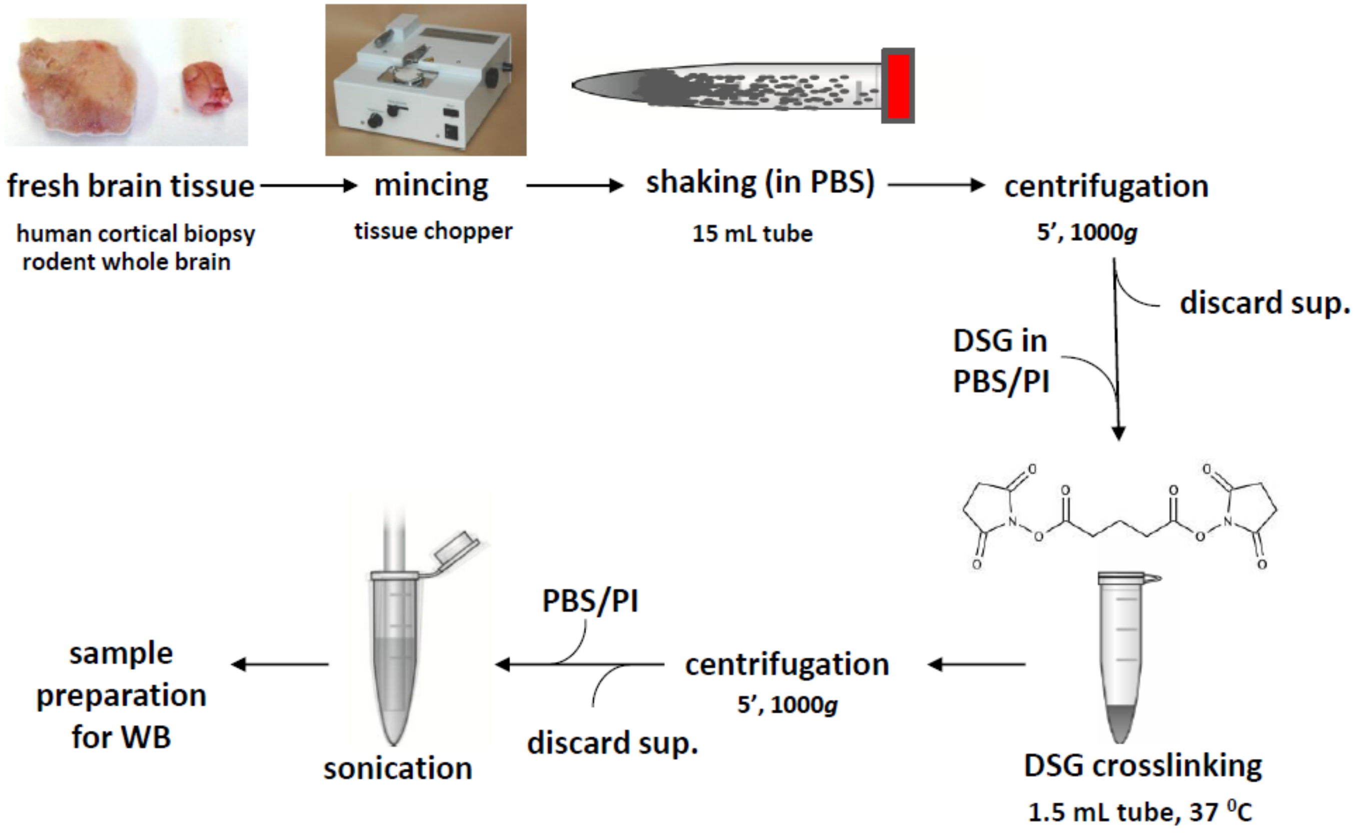

Supplementary Fig. 1: Intact cell crosslinking of fresh brain tissue, schematic. The procedure is described in the Methods section of the main document.

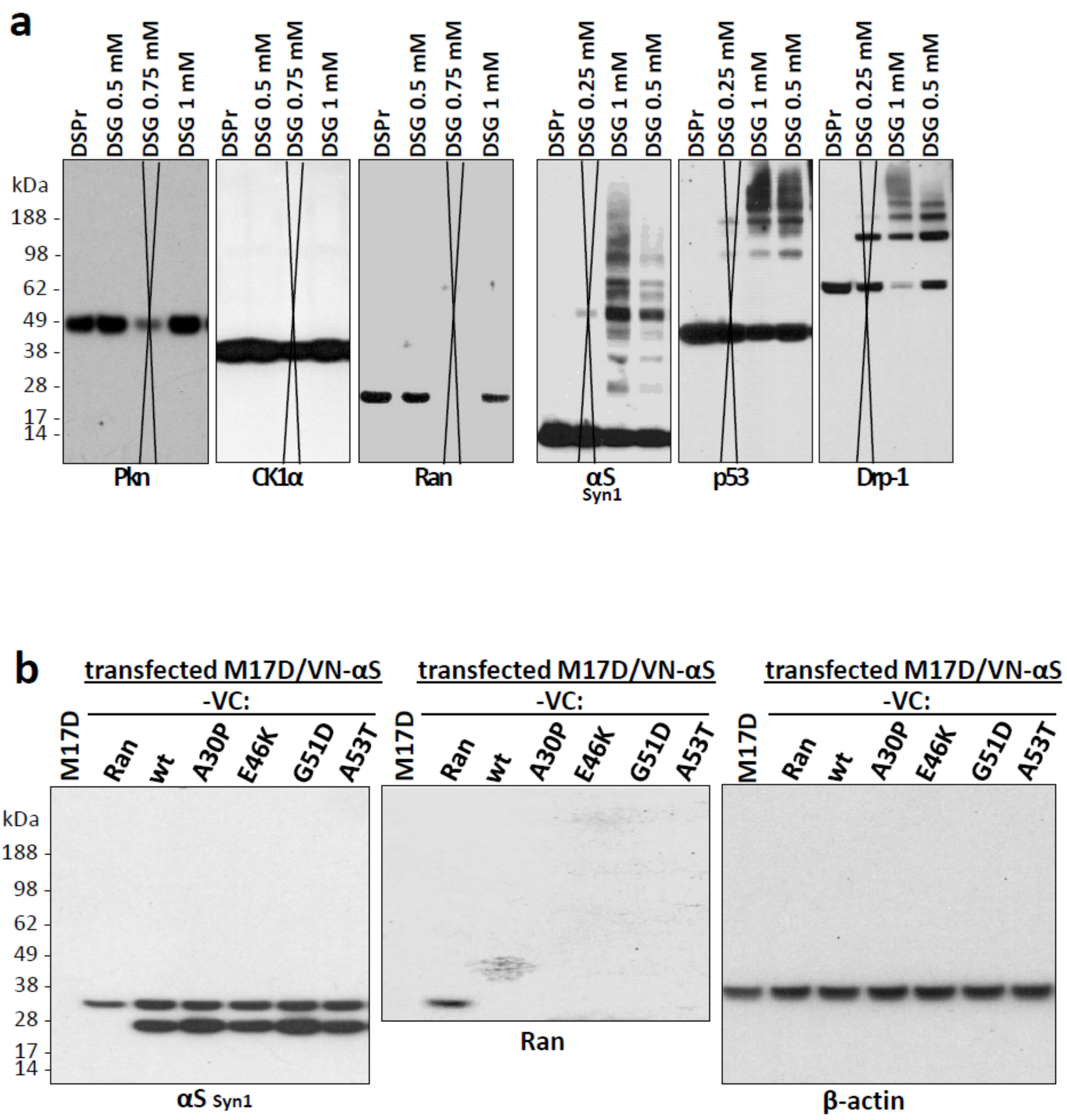

Supplementary Fig. 2. Uncropped scans of the blots shown in Fig. 1. a, Blots corresponding to Fig. 1d. Crossed out are lanes DSG 0.75 mM (insufficient protein content) and DSG 0.25 mM (insufficient crosslinking), which were not used for the final figure. **b,** Blots corresponding to Fig. 1h. A lane of parental M17D cells is shown that is not present inf Fig 1h. Note that in the case of Ran the PVDF membrane had been cut and the lower part developed for a different protein.

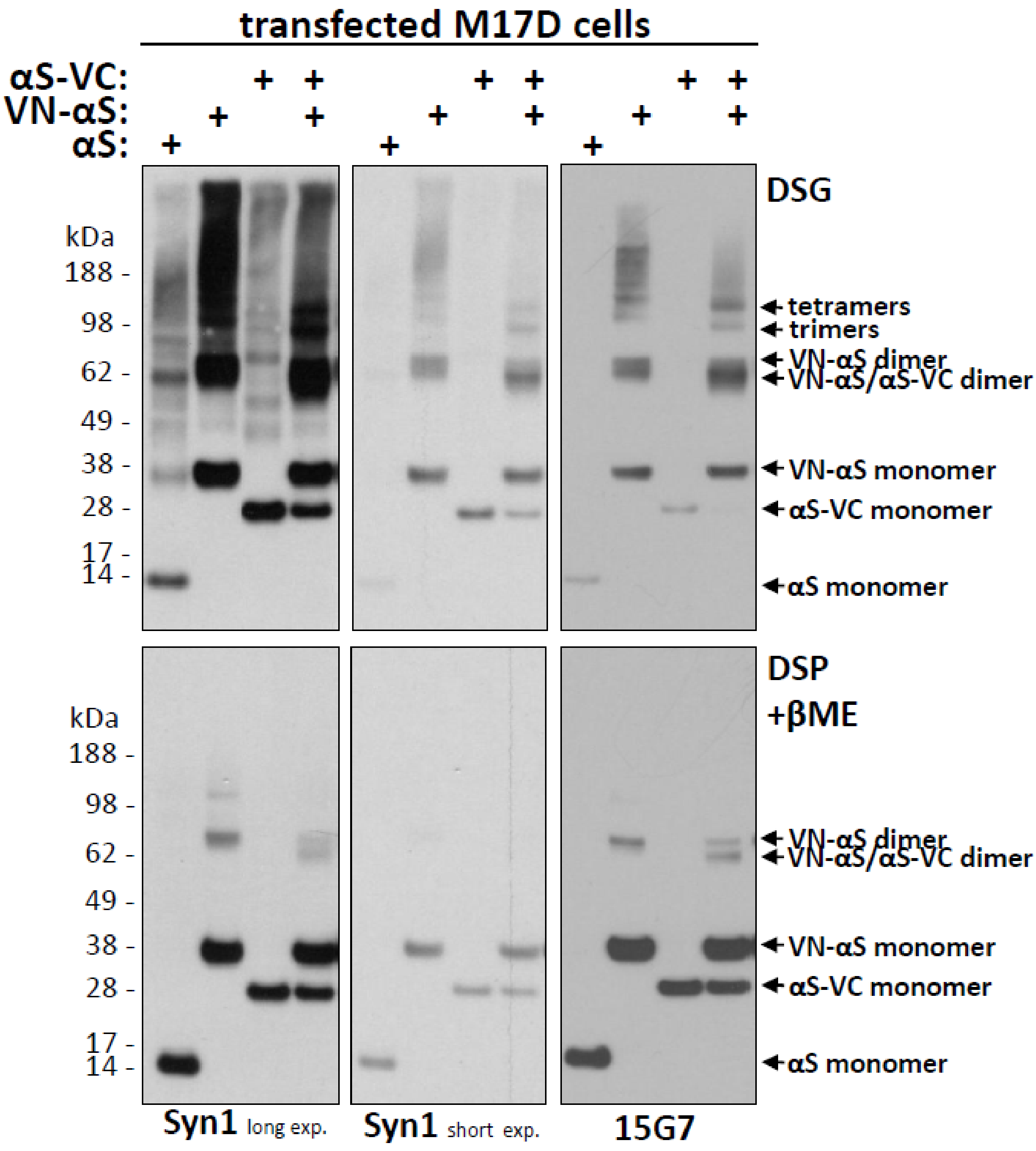

Supplementary Fig. 3: Intact-cell crosslinking analysis of αS Venus YFP complementation constructs. VN-αS (αS N-terminally fused to the N-terminal half of Venus YFP) and αS-VC (αS C-terminally fused to the C-terminal half of Venus YFP) were expressed as indicated in M17D cells for 40 hrs, followed by DSG crosslinking (upper panels) or DSP/βME treatment; for comparison, untagged wt αS (αS) was used. mAbs Syn1 or 15G7 were used for αS detection.

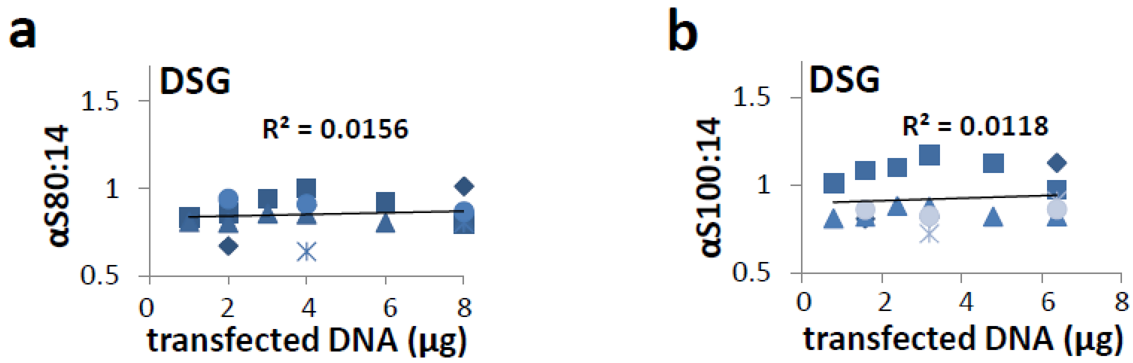

Supplementary Fig. 4: Intact-cell crosslinking of human αS expressed in M17D neuroblastoma cells at variable levels. **a,** αS80:14 ratios for the indicated amounts of transfected DNA (DSG crosslinking). **b,** αS100:14 ratios for the indicated amounts of transfected DNA (DSG crosslinking). Data points generated in the same experiment are indicated by identical symbols ; for details on samples, see Fig. 2a, b, c.

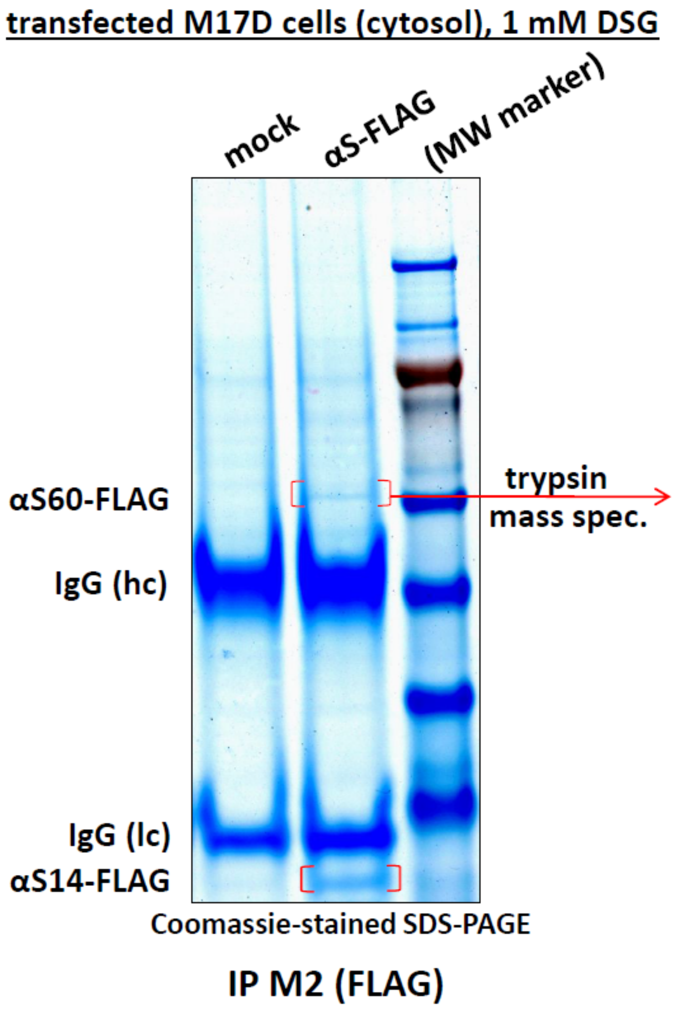

Protein	Coverage	# peptides	Score
αS	51.43	3	88.72
Keratin II	39.29	4	91.17
Hsp-70	32,76	4	177.77
Keratin I	28.41	6	135.43

αS sequences detected:

```
MDVFMKGLSK AKEGVVAAAE
KTKQGVAEAA GKTKEGVLYV
GSKTKEGVVH GVATVAEKTK
EQVTNVGGAV VTGVTAVAQK
TVEGAGSIAA ATGFVKKDQL
GKNEEGAPQE GILEDMPVDP
DNEAYEMPSE EGYQDYEPEA
```

Supplementary Fig. 5: Mass-spectrometric analysis of FLAG-tagged αS60. wt-αS C-terminally fused to a triple-FLAG tag[1] was expressed in M17D cells for 40 hrs followed by DSG crosslinking and FLAG-immunoprecipitation[1], untransfected cells served as a control. Precipitated proteins were run on an SDS-PAGE, which was then Coomassie-stained; hc heavy chain; lc, light chain. The band corresponding to αS60-FLAG was cut out, trypsin-digested in-gel and subjected to mass-spectrometric analysis[2]. Detected proteins (abundance > 1 peptide) were αS, keratin (type I and II) and Hsp-70. Keratins are a common contaminant from sample handling, Hsp-70 had been ruled out as an αS60-component before for several reasons[1] and was presumably precipitated as a non-covalent interactor of monomeric αS14-FLAG (which was present in large amounts due to reduced oligomerization of C-terminally tagged αS, as discussed[1]). Identified αS peptides are underlined.

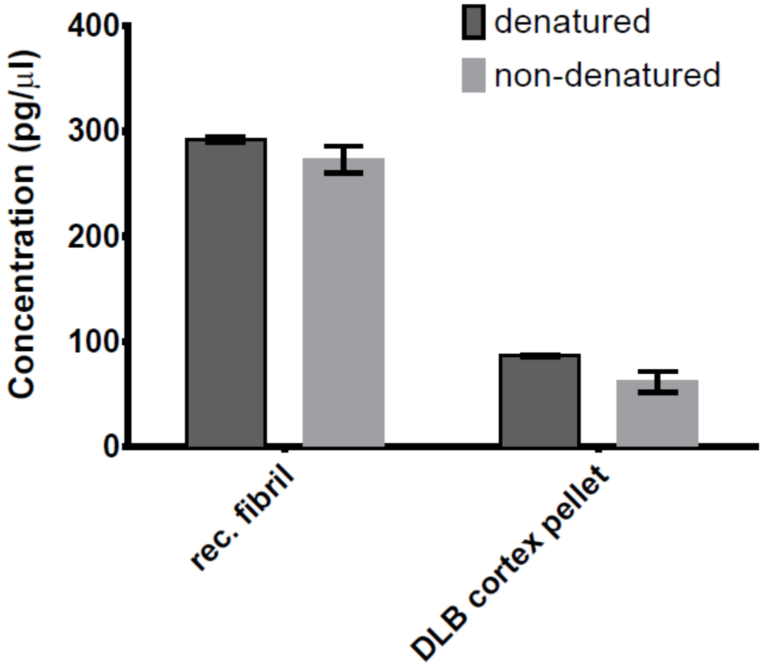

Supplementary Fig. 6: Representative sandwich ELISAs using in-house generated mAbs 2F12 and SOY1 showing equal detection of αS conformers. Samples were pre-aggregated fibrillar recombinant αS (rec. fibril) or partially purified Lewy bodies from the frontal cortex of a DLB patient. Both samples were briefly sonicated and diluted 1:1,000 before measurement. Denatured samples were additionally boiled in 2% SDS before dilution. Error bars = SDs of duplicate experiments.

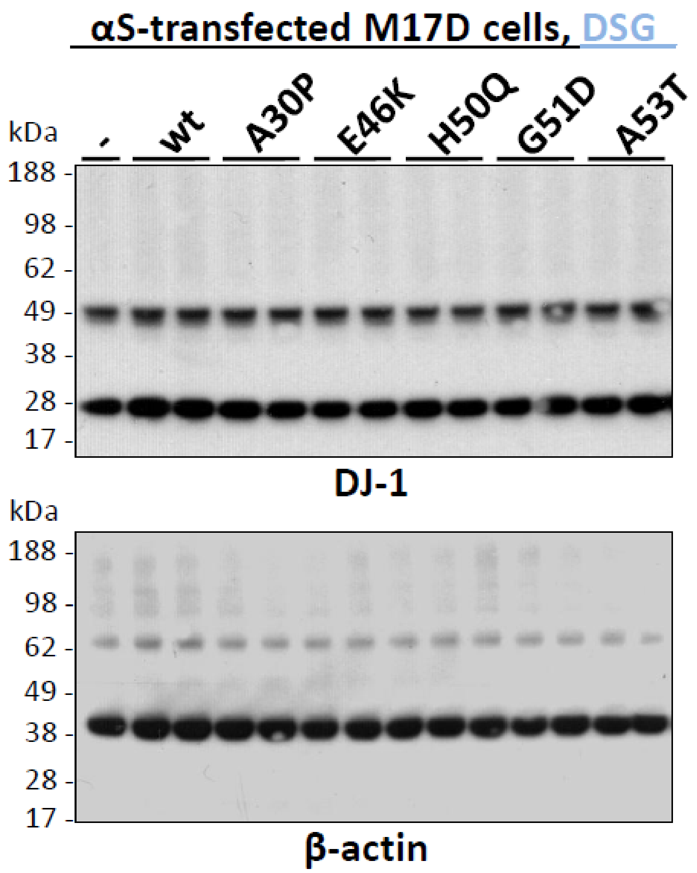

Supplementary Fig. 7. Uncropped scans of the cropped blots shown in Fig. 3. DJ-1 and β-actin blots corresponding to Fig. 3a.

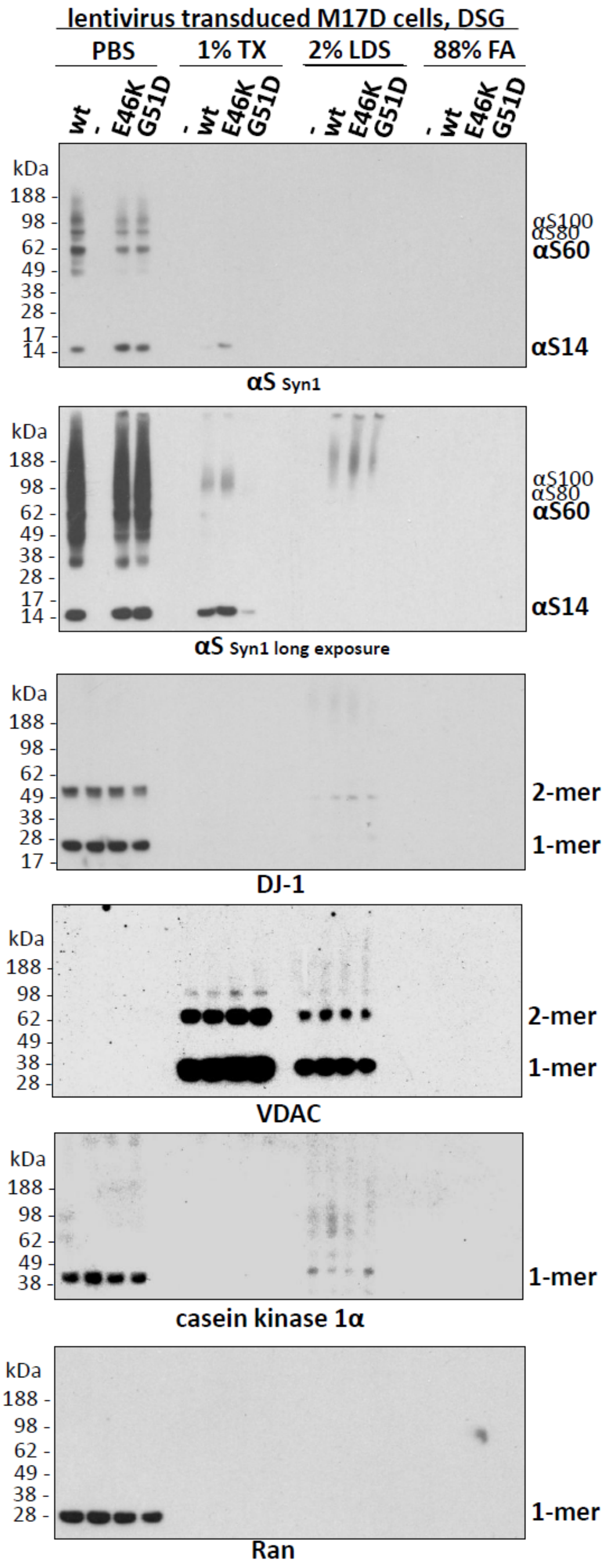

Supplementary Fig. 8. Sequential extraction of stable lentiviral M17D cell pools αS wt, E46K and G51D. αS wt and fPD mutant stable lines as well as non-infected M17D cells (-) after 1mM DSG crosslinking and sequential extraction (PBS → PBS/1%Triton → 2% LDS → 88% formic acid = FA). WB for the indicated proteins. Images are representative of N = 3 experiments performed on different days using the same cell pools.

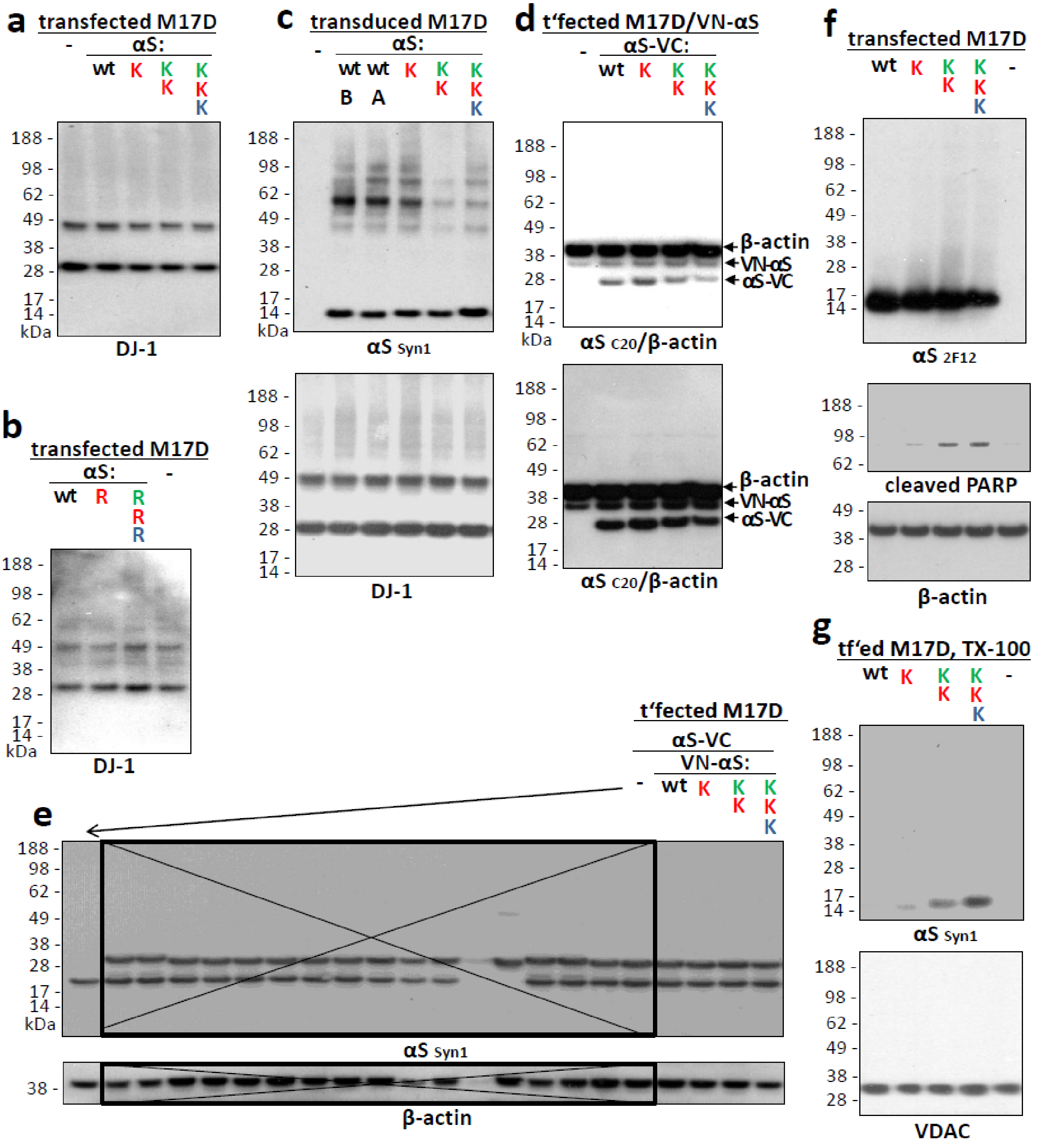

Supplementary Fig. 9. Uncropped scans of the cropped blots shown in Fig. 6. a, DJ-1 blot corresponding to Fig. 6b. **b,** DJ-1 blot corresponding to Fig. 6c. **c,** αS (mAb Syn1) and DJ-1 blots corresponding to Fig. 6d. **d,** αS (pAb C20) and β-actin blot corresponding to Fig. 6e. Note that the two antibodies were mixed and developed together. In Fig. 6e, the shorter exposure (top) was used for β-actin and the longer exposure (bottom) was used for αS. **e,** αS (mAb Syn1) and β-actin blots corresponding to Fig. 6f. Crossed out are samples that were not used in final figure. **f,** αS (mAb 2F12), cleaved PARP and β-actin blots corresponding to Fig. 6g. Cleaved PARP and β-actin were developed on the same PVDF membrane that was cut prior to incubation with antibodies. **g,** αS (Syn1) and VDAC blots corresponding to Fig. 6h.

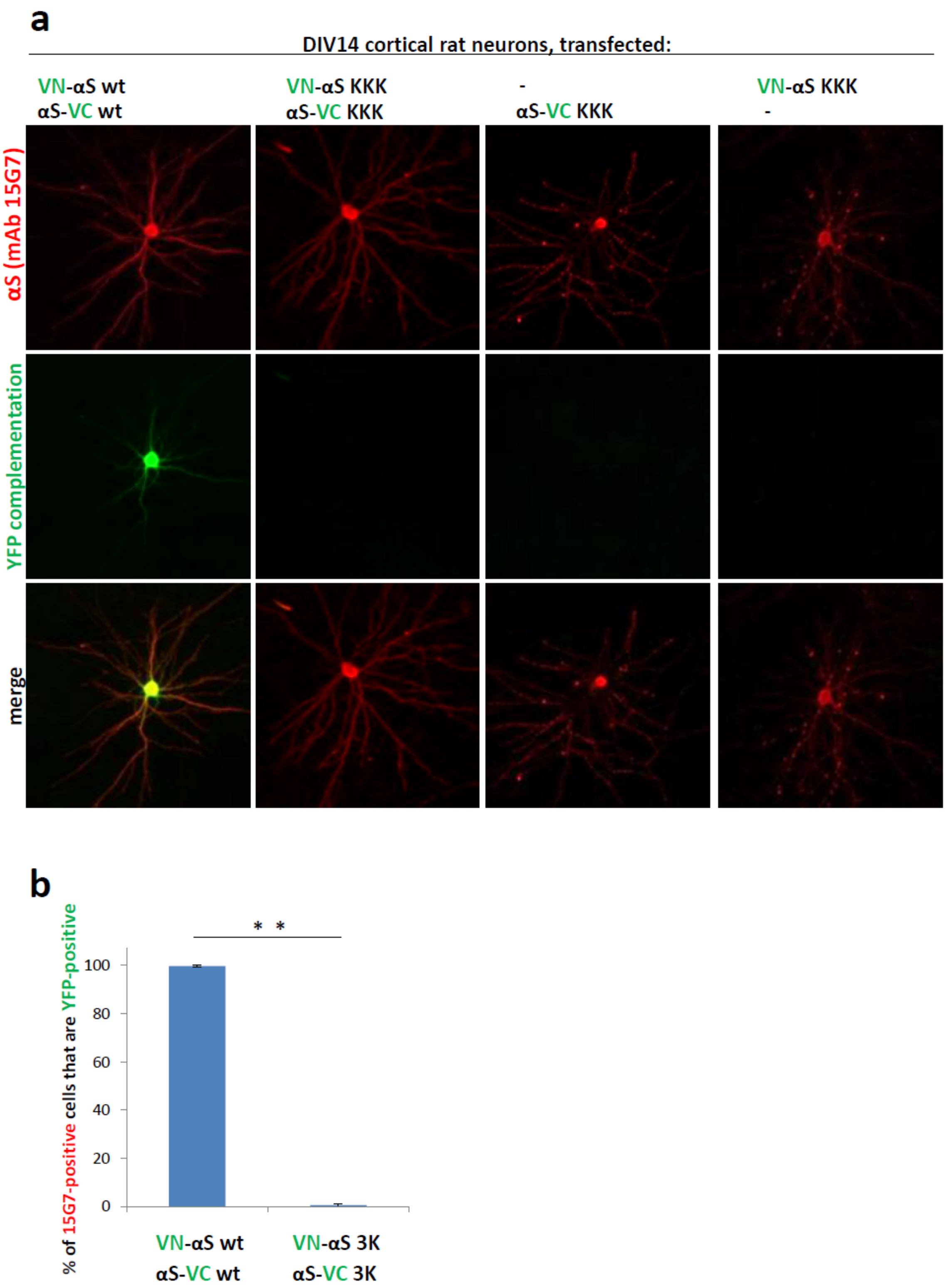

Supplementary Fig. 10. Lack of YFP complementation for aS-KKK variant expressed in primary neurons. a, Fluorescence microscopy and YFP complementation analysis of rat neurons (DIV14) transfected with the indicated untagged αS variant; immunofluorescence with *human-specific* mAb 15G7 (red) and YFP signal (green), plus merge picture. **b,** Percentages of cells clearly positive for 15G7 or YFP were counted blinded (right: N = 3; 100 cells each; $p < 0.01$ Student's t-test).

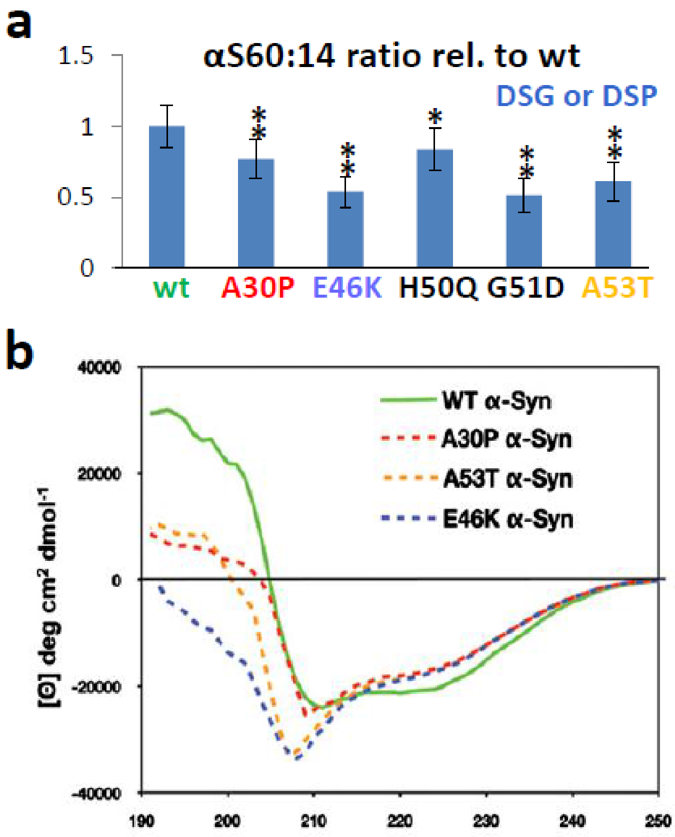

Supplementary Fig. 11. Comparison of αS60:14 ratios for wt and mutant αS (this study, Fig. 3D) to CD data obtained by Wang et al. (see Fig. 1C in [3]). a, αS60:14 ratio determined by DSG or DSP crosslinking analysis for the indicated αS variants (see Fig. 3 for details). **b,** CD spectra of αS wild-type (solid green), A30P (red dashed line), A53T (orange dash), and E46K (blue dash).

Supplementary References

1. Dettmer, U., Newman, A. J., Luth, E. S., Bartels, T. & Selkoe, D. In vivo cross-linking reveals principally oligomeric forms of α-synuclein and β-synuclein in neurons and non-neural cells. *J. Biol. Chem.* **288,** 6371–6385 (2013).
2. Théberge, R., Infusini, G., Tong, W., McComb, M. E. & Costello, C. E. Top-Down Analysis of Small Plasma Proteins Using an LTQ-Orbitrap. Potential for Mass Spectrometry-Based Clinical Assays for Transthyretin and Hemoglobin. *Int. J. Mass Spectrom.* **300,** 130–142 (2011).
3. Wang, W. *et al.* A soluble α-synuclein construct forms a dynamic tetramer. *Proc. Natl. Acad. Sci. U. S. A.* **108,** 17797–17802 (2011).
